# Binding-affinity predictions of HSP90 in the D3R Grand Challenge 2015 with docking, MM/GBSA, QM/MM, and free-energy simulations

**DOI:** 10.1007/s10822-016-9942-z

**Published:** 2016-08-26

**Authors:** Majda Misini Ignjatović, Octav Caldararu, Geng Dong, Camila Muñoz-Gutierrez, Francisco Adasme-Carreño, Ulf Ryde

**Affiliations:** 1 Department of Theoretical Chemistry, Lund University, Chemical Centre, P. O. Box 124, 221 00 Lund, Sweden; 2 Centro de Bioinformática y Simulación Molecular, Facultad de Ingeniería, Universidad de Talca, 2 Norte 685, Talca, Chile

**Keywords:** Ligand-binding affinity, Induced-fit docking, MM/GBSA, QM/MM, Big-QM, Free-energy perturbation, Continuum solvation, Bennett acceptance ratio, D3R grand challenge, Blind-test competition

## Abstract

We have estimated the binding affinity of three sets of ligands of the heat-shock protein 90 in the D3R grand challenge blind test competition. We have employed four different methods, based on five different crystal structures: first, we docked the ligands to the proteins with induced-fit docking with the Glide software and calculated binding affinities with three energy functions. Second, the docked structures were minimised in a continuum solvent and binding affinities were calculated with the MM/GBSA method (molecular mechanics combined with generalised Born and solvent-accessible surface area solvation). Third, the docked structures were re-optimised by combined quantum mechanics and molecular mechanics (QM/MM) calculations. Then, interaction energies were calculated with quantum mechanical calculations employing 970–1160 atoms in a continuum solvent, combined with energy corrections for dispersion, zero-point energy and entropy, ligand distortion, ligand solvation, and an increase of the basis set to quadruple-zeta quality. Fourth, relative binding affinities were estimated by free-energy simulations, using the multi-state Bennett acceptance-ratio approach. Unfortunately, the results were varying and rather poor, with only one calculation giving a correlation to the experimental affinities larger than 0.7, and with no consistent difference in the quality of the predictions from the various methods. For one set of ligands, the results could be strongly improved (after experimental data were revealed) if it was recognised that one of the ligands displaced one or two water molecules. For the other two sets, the problem is probably that the ligands bind in different modes than in the crystal structures employed or that the conformation of the ligand-binding site or the whole protein changes.

**Electronic supplementary material:**

The online version of this article (doi:10.1007/s10822-016-9942-z) contains supplementary material, which is available to authorized users.

## Introduction

One of the prime challenges of computational chemistry is to predict the free energy for the binding of small molecules to biomacromolecules. Many biological functions are exerted by the binding of substrates or inhibitors to enzymes or effectors to receptors, and the prime aim of drug development is to find small molecules that bind strongly to the target receptor, but with a small effect on other biosystems. Consequently, much effort has been spent to develop methods with this aim, ranging from simple docking and scoring approaches, via end-point methods, such as MM/GBSA (molecular mechanics combined with generalised Born and solvent-accessible surface area solvation) and linear interaction energies (LIE), to strict free-energy simulation (FES) methods [1–4].

Numerous studies have evaluated the performance of various binding-affinity methods, e.g. docking [5, 6], MM/GBSA [7, 8], and FES methods [9–11]. The conclusion has typically been that docking methods can rapidly find the correct binding pose among several other poses, but that they have problems to correctly rank the affinities of a set of ligands to the same protein. MM/GBSA calculations typically give a better ranking of the ligands and an understanding of energy terms involved in the binding, but often vastly overestimate energy differences and the results strongly depend on the employed continuum-solvation model [2, 12]. Large-scale tests of FES calculations have given rather impressive results for relative binding affinities of similar ligands to the same protein, with mean absolute deviations (MAD) of 4–6 kJ/mol [9–11]. However, the comparisons have been primarily directed to small changes in the ligands and the performance is uneven, with very good results for some proteins, but quite poor performance for other proteins, occasionally with errors of over 20 kJ/mol.

Comparisons of different approaches for the same test case are less common and often half-hearted in the meaning that the authors are experts or developers of one approach and include other methods mainly to show that they are worse [10, 13, 14]. In this respect, blind-test competitions are important to judge the true performance of different approaches, allowing experts to provide predictions that are not biased by the experimental results. In the SAMPL4 octa-acid host–guest challenge for binding affinities, FES methods gave the best results (the root-mean-squared deviation, RMSD, was 5 kJ/mol and the correlation coefficient, *R* ^2^, was 0.9), although docking gave results of only slightly worse quality (RMSD = 6 kJ/mol, *R* ^2^ = 0.8) [15–17]. However, this test case was ideal for FES calculations with quite small differences between the ligand and a conserved net charge. For the cucurbit [7] uril host, the results were worse and more varying, but a FES-based approach still gave the best results RMSD = 12 kJ/mol, *R* ^2^ = 0.8, whereas docking gave poor results (RMSD = 33 kJ/mol, *R* ^2^ = 0.1) [15, 17]. The results for the SAMPL3 host–guest systems were even worse, with either RMSD and *R* ^2^ both low, e.g. 6 kJ/mol and 0.4 for the MM/GBSA-like solvent interaction energy (SIE) approach [18], or both high, e.g. 47 kJ/mol and 0.8 for FES [19].

For protein systems, the results have been even worse. For the HIV integrase binding-affinity challenge in SAMPL4, a SIE approach was pointed out as best with a mean absolute deviation (MAD) of 5 kJ/mol, but it gave a negative correlation (*R* = −0.3) [20, 21]. Docking calculations gave positive correlation (*R* = 0.5–0.6), but the MAD was high (76–113 kJ/mol), because a raw docking score was employed [22]. An MM/PBSA approach gave a lower MAD, 16 kJ/mol, and a positive correlation (*R* = 0.4) [20]. The reason for these poor results was that all eight experimental binding affinities were within 4 kJ/mol.

A similar problem applied to the trypsin challenge in SAMPL3, where the experimental range of the 17 ligands was only 9 kJ/mol (and 13 within 4 kJ/mol). Unfortunately, no overview article was published for this test case, so it is hard to reach any unbiased conclusions. A comparison of five methods indicated that none of them gave any useful correlation (*R* ^2^ < 0.02), but LIE gave a correct ranking of all ligands for which both the experimental and computational estimates were statistically significant [14]. Docking with the Glide software gave the lowest MAD (3 kJ/mol) and also the best discrimination between binders and non-binders (the area under the receiver-operating-characteristic curve, AUC, was 0.8). LIE gave a slightly larger MAD (4 kJ/mol), but a poorer-than-random AUC (0.3). MM/PBSA and MM/GBSA gave large MAD (20 and 16 kJ/mol), but reasonable AUC (0.7).

In this article, we present a comparison of four different approaches to calculate absolute or relative binding affinities for three sets of similar ligands to the heat-shock protein 90 (HSP90) within the drug-design data resource (D3R) 2015 grand challenge [23]. HSP90 is a conserved chaperone protein that is expressed ubiquitously in high concentration [24], in particular in cancer cells [25, 26] and therefore of large interest as a multiple-oncogenic-pathway therapeutics [27–30]. We have performed docking with the Glide software [31], MM/GBSA scoring with single minimised structures with the Prime software [32], and FES calculations of relative affinities. In addition, we have made an attempt to perform combined quantum and molecular mechanics (QM/MM) scoring with an approach similar to that developed by Grimme and coworkers for host–guest systems [33, 34] combined with our big-QM approach to obtain stable QM/MM energies for proteins [35].

## Methods

**Fig. 1:**
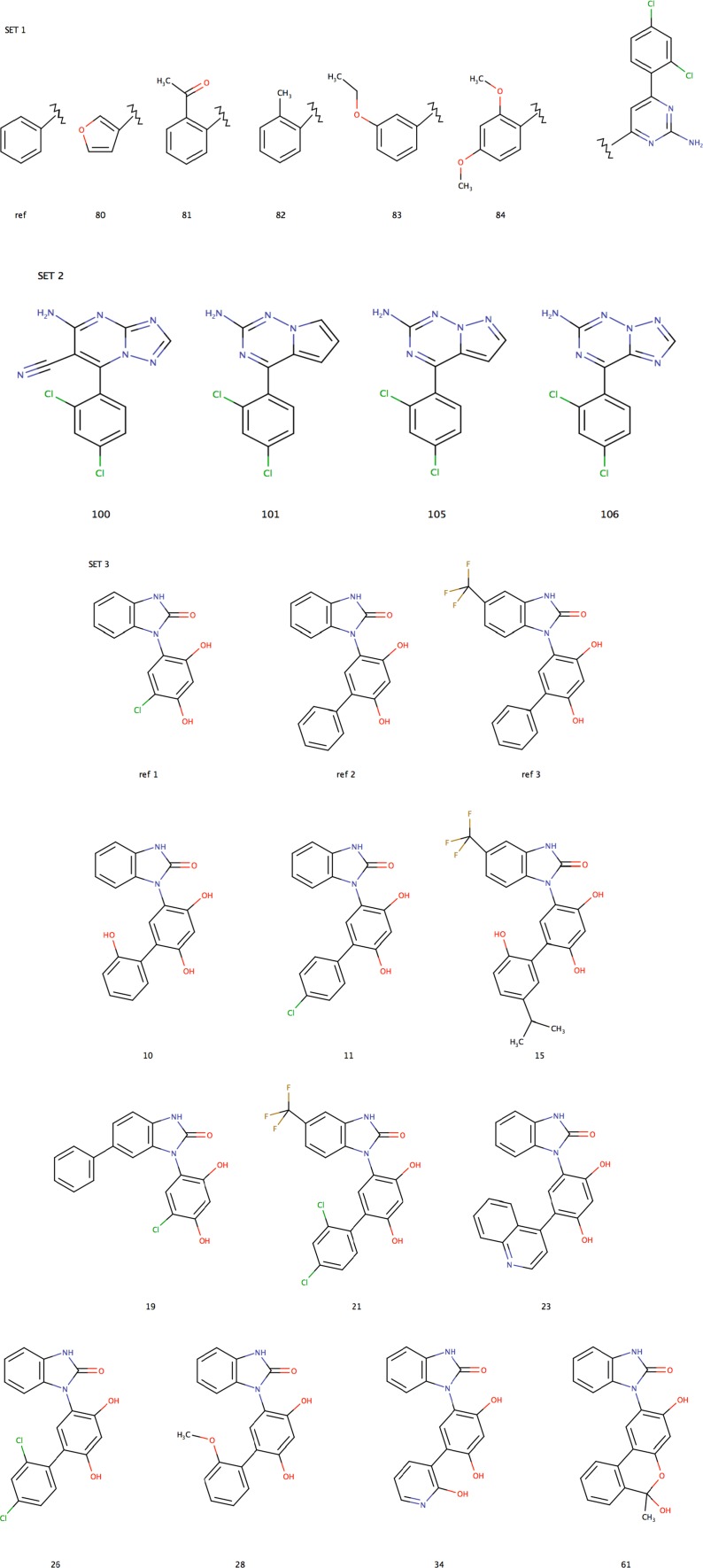
Structures of all ligands from sets 1, 2, and 3, considered in this study. The additional reference ligands that were employed for sets 1 and 3 are also shown. The numbering of ligands is the same as in the HSP90 D3R grand challenge data set. Ligands of sets 1 and 3 are shown in conformation 1

Relative binding free energies for three sets of ligands binding to HSP90 were estimated as a part of the D3R Grand Challenge 2015 [23]. Sets 1, 2, and 3 consist of five, four, and ten ligands, respectively and involve chemically similar ligands, which allow for the calculation of relative binding free energies by alchemical FES methods. The 19 ligands are shown in Fig. 1. The FES calculations employed four additional reference ligands, which are also shown in the figure. Four methods were used to estimate the binding affinities, viz. docking, MM/GBSA, QM/MM, and FES. They are described in separate sections below.

The studies were based on five protein crystal structures (PDB files 3VHA [36], 2WI7 [37], 3FT5 [38], 3OW6 [39], and 4YKR [40]), which are described in Table 1. They were selected based on the quality of the structure, the conformation of the entrance of the ligand-binding pocket (closed, semi-closed, or open [38]) and the similarity of the co-crystallised ligand with the ligands in the various sets. The ligands in the crystal structures are shown in Figure S1 in the supplementary material. The 3VHA structure was obtained at 1.4 Å resolution and it contains a ligand that is quite similar to those in set 1. It was the only structure used for the set 1 calculations and it was also used for some set 2 calculations. However, the ligand in 2WI7 is more similar to the set 2 ligands, although the resolution is rather poor, 2.5 Å. The ligand in 3FT5 is also similar to the set 2 ligands, but it is much smaller and the binding pocket is in the closed conformation. The resolution is intermediate (1.9 Å). For set 3, two structures were employed, 3OW6 and 4YKR. They are of similar resolution (1.8 and 1.6 Å, respectively) and contain similar ligands of a proper scaffold (the ligand is slightly smaller in the 3OW6 structure).

**Table 1:** Description of the protein structures used in this study and protonation states of the His residues

Crystal structure	Resolution (Å)	State	His protonation				Set	Ref.
			77	154	189	210		
3VHA	1.39	Semi-closed	HIP	HIP^a^	HIP	HIE	1, 2	[36]
2WI7	2.50	Open	HIP	HIE	HIP	HIE	2	[37]
3FT5	1.90	Closed	HIP	HIE	HIP	HIE	2	[38]
3OW6	1.80	Semi-closed	HIP	HID	HIP	HIE	3	[39]
4YKR	1.61	Closed	HIP	HIE	HIP	HIE	3	[40]

^a^HID in the docking and QM/MM calculations

### Docking calculations

The docking calculations were set up with the Schrödinger 2015-2 suite of software [41]. They were based on the 3VHA [36] structure for set 1 and 2, and the 3OW6 [39] structure for set 3. The 4YKR [40] structure was also tested for set 3, but no reasonable docked structures could be obtained for ligands **15** and **61**. After the experimental results were revealed, docking calculations were also performed with the 2WI7 crystal structures for set 2 [37]. The protein preparation wizard module was employed for preparing the protein structures [41]. Crystal water molecules more than 5 Å away from the ligand were removed prior to the hydrogen-bond optimisation and protein minimisation stages. The hydrogen-bond network was optimised at pH 7 by sampling Asn and Gln rotamers, hydroxyls, thiols, and water orientations. The protonation states for Asp, Glu, and His were derived from PropKa 3.1 [42, 43]. The protonation states employed for the His residues are shown in Table 1.

According to the recommended protein preparation protocol [44], the prepared structures were then relaxed by means of a restrained molecular minimisation using the Impact refinement module using the OPLS 2005 force field [45], with heavy atoms restrained to remain within a RMSD of 0.30 Å from the initial coordinates. This allows hydrogen atoms to be freely minimised and heavy atoms can move to relax strained bonds, angles, and steric clashes. After a closer inspection of the hydrogen-bond network in the ligand-binding site, three (3OW6) or four (3VHA and 2WI7) water molecules were identified that form at least one hydrogen bond to either the protein or the ligand. These water molecules were kept in the calculations, whereas the remaining crystal water molecules were deleted. For set 2, one of the four crystal-water molecules (called Wat2 below) made steric clashes with one of the ligands. In the calculations with the 3VHA structure, this water molecule was deleted when docking all four ligands, whereas with the 2WI7 structure, Wat2 was deleted only for ligand **100** and was kept for the other three ligands.

The ligand structures were built using the Maestro visualisation software [46] and then prepared with the LigPrep module [47], in which the ionisation and tautomeric states at pH 7 were predicted using Epik [48]. Finally, an energy minimisation in gas phase using Macromodel [49] with the OPLS 2005 force field [45] was performed.

All docking calculations were performed using the Glide software [31]. Initial docking studies using the standard-precision (SP) mode with default parameters for grid and pose generation were unable to produce poses that fitted into the binding site for the tested inhibitors, probably because the binding cavity is too tight to fit molecules larger than the co-crystallised ligands. Scaling down the van der Waals radii of non-polar protein atoms, a crude approach to allow steric clashes during docking, did not produce better results. Therefore, we employed the induced-fit docking (IFD) workflow [50, 51] to generate alternative conformations of the receptor suitable to bind the studied ligands, by allowing the protein to undergo sidechain or backbone movements during the docking.

The IFD procedure has four steps: (1) initial Glide docking using a softened-potential (van der Waals scaling of 0.5) into a rigid receptor to generate an ensemble of poses; (2) sampling of protein conformations using the sidechain prediction module Prime [32], followed by a structure minimisation of each protein–ligand complex; (3) redocking of the ligands into low energy induced-fit structures from the previous step using default Glide settings (no scaling of van der Waals interactions); and (4) estimation of the binding energy of the optimised protein–ligand complexes.

The IFD standard protocol was employed, generating up to 20 poses per ligand on each iteration. The docking grid was generated for the co-crystallised ligands. The OPLS 2005 force field [45] was used for the minimisation stage, in which residues within 5 Å of each ligand pose were optimised. Pose rescoring was performed with the SP docking mode. All other parameters were set to their default values. Finally, the obtained docking poses were visually inspected, filtering out those that did not adopt a similar position and orientation as the reference inhibitors. Only the most favourable docking pose for each ligand was selected for structural analysis.

### Pose rescoring with MM/GBSA

All docking poses were rescored with the MM/GBSA approach, as implemented in the Prime program in the Schrödinger software suite [32, 41]. It employed a single minimised protein–ligand structure, thus establishing an efficient approach to rapidly refine and rescore docking results. We employed the variable dielectric solvent model VSGB 2.0 [52], which includes empirical corrections for modelling directionality of hydrogen-bond and π-stacking interactions. This approach has been shown to give good binding free energies for a wide range of protein–ligand complexes [53]. Residues within 5.0 Å of the ligand were allowed to relax during the MM minimisation of the complex, keeping the rest of the structure fixed.

### QM/MM scoring

The docked structures were also rescored using a QM/MM approach, developed as a combination of the QM-cluster approach for the study of the binding in host–guest systems by Grimme and coworkers [33, 34] and the big-QM approach developed in our group to obtain stable QM/MM energies in proteins [35]. The QM/MM calculations employed the docked structures, but the first four residues in the protein for sets 1 and 2 were deleted (Pro11–Glu14, because they are hanging free in solution, without any interactions with the remainder of the protein) and a MOPS buffer molecule, far from the ligand-binding site, was also deleted. The docked structure was solvated in a sphere of water molecules with a radius of 37 Å, centred on the geometric centre of the protein, giving a total of ~18,600 atoms. Hydrogen atoms and water molecules were optimised with a 120 ps simulated annealing calculation with an initial temperature of 370 K, followed by a minimisation using the Amber software [54].

#### QM/MM calculations

**Fig. 2:**
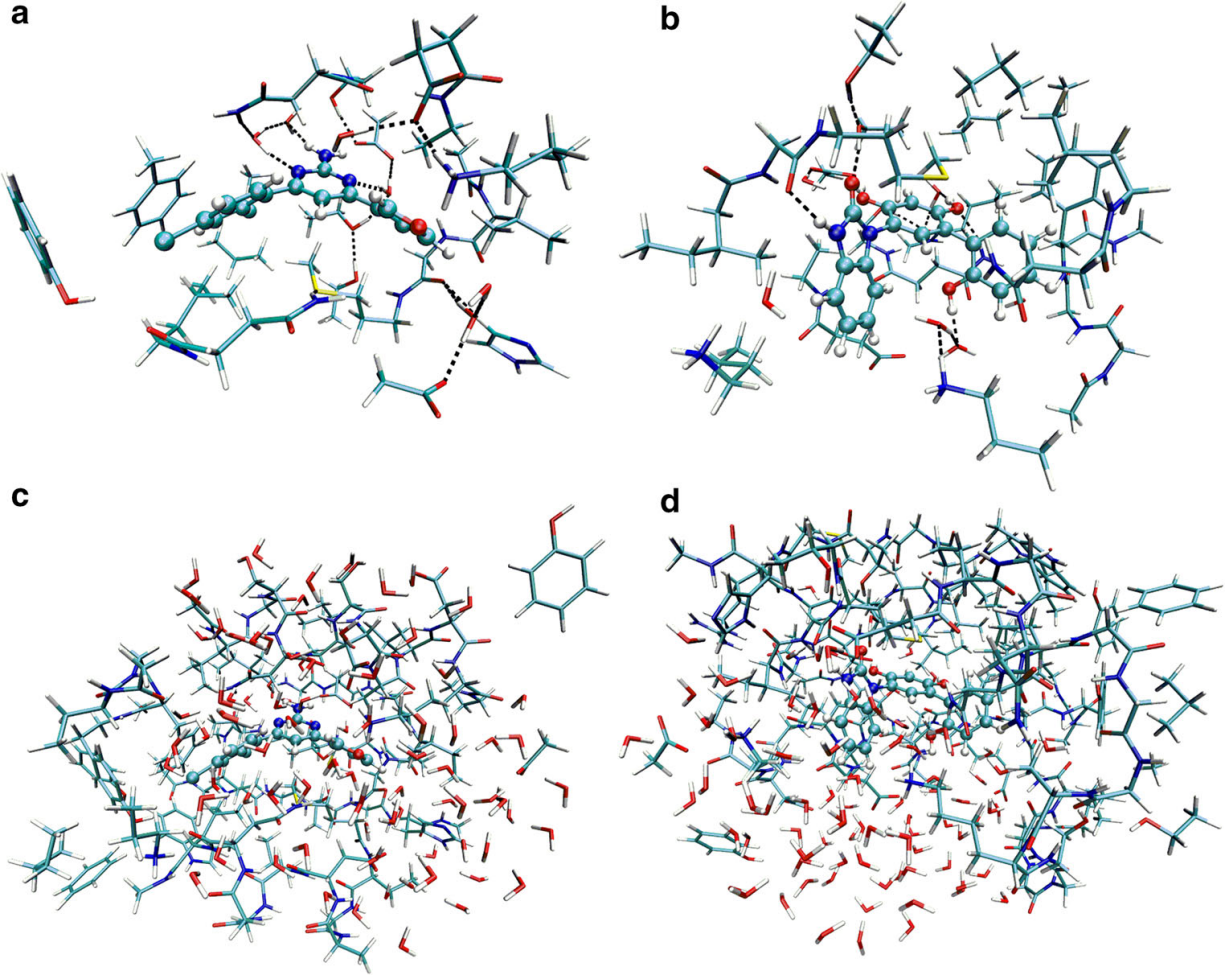
The QM systems used in the QM/MM optimisations for sets 1 and 2 (**a**), and set 3 (**b**), as well as in the big-QM calculations (**c**, **d**). The ligand is shown in *ball*-and-*sticks* representation

The QM/MM calculations were performed with the ComQum software [55, 56]. In this approach, the protein and solvent are split into two subsystems: System 1 (the QM system) was relaxed by QM methods. For sets 1 and 2, it consisted of the ligand, as well as Asn51, Ser52, Asp54, Ala55, Lys58, Asp93, Gly95, Ile96, Gly97, Met98, Asp102, Asn106, Leu107, Phe138, Tyr139, Val150, Thr152, His154, Thr184, and Val186. For set 3, the QM system included residues Leu48, Ile49, Asn51, Ser52, Asp54, Ala55, Lys58, Asp93, Ile96, Gly97, Met98, Asn106, Leu107, Lys112, Gly135, Val136, Gly137, Phe138, Tyr139, Val148, Val150, Thr152, Thr184, and Val186. In both cases, the six water molecules closest to the ligand were also included, giving a total of ~280 and ~320 atoms, respectively. The two QM systems are shown in Fig. 2a, b. System 2 consisted of the remaining part of the protein and the solvent. It was kept fixed at the original docked coordinates.

In the QM calculation, System 1 was represented by a wavefunction, whereas all the other atoms were represented by an array of partial point charges, one for each atom, taken from MM libraries. Thereby, the polarisation of the QM system by the surroundings is included in a self-consistent manner (electrostatic embedding). When there is a bond between systems 1 and 2 (a junction), the hydrogen link-atom approach was employed: the QM system was capped with hydrogen atoms (hydrogen link atoms, HL), the positions of which are linearly related to the corresponding carbon atoms (carbon link atoms, CL) in the full system [55, 57]. All atoms were included in the point-charge model, except the CL atoms [58].

The total QM/MM energy in ComQum is calculated from [55, 56]

1 $${E_{\text{QM/MM}} = E_{\text{QM1 + ptch2}}^{\text{HL}} + E_{{{\text{MM12,q}}_{ 1} { = 0}}}^{\text{CL}} - E_{{{\text{MM1,q}}_{ 1} { = 0}}}^{\text{HL}} }$$

where ${E_{\text{QM1 + ptch2}}^{\text{HL}} }$ is the QM energy of the QM system truncated by HL atoms and embedded in the set of point charges modelling system 2 (but excluding the self-energy of the point charges). ${E_{MM 1 ,q 1= 0}^{HL} }$ is the MM energy of the QM system, still truncated by HL atoms, but without any electrostatic interactions. Finally, ${E_{MM 1 2 ,q 1= 0}^{CL} }$ is the classical energy of all atoms in the system with CL atoms and with the charges of the QM system set to zero (to avoid double counting of the electrostatic interactions). By this approach, which is similar to the one used in the ONIOM method [59], errors caused by the truncation of the QM system should cancel.

The geometry optimisations were continued until the energy change between two iterations was less than 2.6 J/mol (10^−6^ a.u.) and the maximum norm of the Cartesian gradients was below 10^−3^ a.u. The QM calculations were carried out using Turbomole 7.0 software [60]. The geometry optimisations were performed using the TPSS [61] functional in combination with def2-SV(P) [62] basis set, including empirical dispersion corrections with the DFT-D3 approach [63]. The MM calculations were performed with the Amber software [54], using the Amber ff14SB force field [64].

#### Big-QM calculations

Previous studies have shown that QM/MM energies strongly depend on the size of the studied QM system [58, 65]. To avoid this problem, we have developed the big-QM approach to obtain converged energies [35]: we constructed a very large QM system, consisting of all residues with at least one atom within 7.5 Å of the ligand in any of the studied structures. Thus, the QM system was the same for all ligands. For sets 1 and 2 residues 22, 26, 47–59, 61, 62, 78, 91–108, 112, 135–139, 141, 142, 148–155, 162, 180, and 182–187, as well as the 79 closest water molecules were included, in total ~970 atoms. For the set 3 ligands, the QM system consisted of residues 22, 26, 29, 44, 45, 47–59, 61, 62, 77, 78, 90–99, 102–113, 115, 131–142, 148–155, 162, 180, and 182–188, as well as the 80 closest water molecules, in total ~1160 atoms. Both systems included the single buried charged group in the protein, Asp93. The ligand is not covalently connected to the protein, so it does not form any junction to the protein (in the standard big-QM approach, all buried charges in the protein should be included and junctions should be moved two residues away from the minimal QM system [35]). The QM systems are shown in Fig. 2c, d. The big-QM calculations were performed on coordinates from the QM/MM optimisation. Two sets of big-QM calculations were performed. In the first, a point-charge model of the surroundings was included, because this gave the fastest calculations in our previous tests [35]. In the second approach, we performed the calculation without the point-charge model, but included instead a conductor-like screening model (COSMO) [66, 67] continuum solvent with a dielectric constant of 80. In both cases, the calculations were performed at the TPSS/def2-SV(P) level of theory and they employed the multipole-accelerated resolution-of-identity J approach [68].

#### Additional energy terms

To the big-QM energy, we added the DFT-D3 dispersion correction, calculated for the same big-QM system with Becke–Johnson damping [69], third-order terms, and default parameters for the TPSS functional using dftd3 program [70].

Moreover, we added a correction for increasing the basis set from def2-SV(P) to def2-QZVP [71], calculated for the QM system used in the QM/MM geometry optimisations with the TPSS method and including a point-charge model of the surroundings:

2 $$\Delta E_{\text{bsc}} = E\left( {{\text{TPSS/def2-QZVP}}} \right){-}E\left( {{\text{TPSS/def2-SV(P)}}} \right)$$

Thermal corrections to the Gibbs free energy at 298 K and 1 atm pressure (*G*_therm_; including zero-point vibrational energy (ZPE) entropy, and enthalpy corrections) were calculated by an ideal-gas rigid-rotor harmonic-oscillator approach [72] from vibrational frequencies calculated at the MM level. These were obtained for truncated systems in which only residues and water molecules within 12 Å of the ligand were included in the calculations. Moreover, residues and water molecules more than 8 Å from the ligand were kept fixed in the calculations and they were ignored when the frequencies were calculated. Such an approach is employed in MM/PBSA calculations [73] and it has been found to give reliable results [74]. To obtain more stable results, low-lying vibrational modes were treated by the free-rotor approximation, using the interpolation model suggested by Grimme and ω_0_ = 100 cm^−1^ [33].

For all energy terms, interaction energies were calculated, i.e. separate calculations were performed for the complex, for the protein without the ligand, and for the isolated ligand:

3 $$\Delta E_{\text{int}} = E\left( {\text{complex}} \right){-}E\left( {\text{protein}} \right){-}E\left( {\text{ligand}} \right)$$

The protein calculations were always done using the geometry of the complex after removal of the ligand. For the free ligand, we did two sets of calculations. The first was single-point calculations on the QM/MM structures of the complex, whereas in the second approach, we optimised the geometry of the ligand at the TPSS/def2-SV(P) level of theory in a COSMO continuum solvent with a dielectric constant of 80. This allowed for the calculation of the relaxation energy of the ligand (i.e. the difference in the TPSS/def2-QZVP energy of ligand when optimised in the complex or isolated in the COSMO solvent).

Several approaches were tested to calculate the solvation energy of the complex. In particular, we tested the QM/MM-PBSA and -GBSA approaches [75], using Poisson–Boltzmann (PB) or generalised Born (GB) solvation energies of the whole protein–ligand complex after removal of the water molecules. However, this gave strongly varying energies with large differences between the PB and GB results. Therefore, we decided to simply use big-QM calculations performed in a COSMO solvent with a dielectric constant of 80. Such calculations were performed on both the complex and the protein without the ligand. More accurate solvation energies of the ligand (including also non-polar effects) were calculated with the COSMO-RS (real solvent) approach [76, 77] using the COSMOTHERM software [78]. These calculations were based on two single-point QM calculations at the BP/TZVP level of theory, either in vacuum and with an infinite dielectric constant.

Consequently, the final binding free energies involved six energy terms: the big-QM energies in the COSMO solvent, the basis-set correction, the DFT-D3 dispersion energy, the ∆*G*_therm_ free-energy corrections, the relaxation energy of the ligand, and the solvation free-energy correction for the ligand:

4 $$\Delta G_{\text{bind}} =\Delta G_{\text{BQ}} +\Delta E_{\text{bsc}} +\Delta E_{\text{disp}} +\Delta G_{\text{therm}} +\Delta E_{\text{L,rlx}} + {\Delta \Delta }G_{{{\text{L}},{\text{solv}}}}$$

### FES calculations

Relative binding free energies were also estimated by FES calculations. These were set up independently, using slightly different methods. For set 1, the 3VHA structure was used [36], whereas for set 2, two crystal structures were employed: 2WI7 and 3FT5 [37, 38]. The ligand pose in 3FT5 is rotated 180° around C–NH_2_ bond relative to that in 2WI7. We also tried to start the simulations from the protein structure of 3FT5, but with the ligand in the orientation found in structure 2WI7 (3FT5/2WI7). For set 3, the 4YKR structure was used [40]. The structures were protonated using the leap module of Amber 14 [54]. The protonation of His residues was determined by investigating the surroundings, the hydrogen-bond network and the solvent accessibility of each residue (Table 1). The assignment agreed for three of the His residues in all structures. However, for His154, we used a varying assignment, because the crystal structures show that the N^δ1^ atom interacts either with the backbone O atom of Asn155 or the backbone N atom of Asp156. In the 3VHA structure this residue is solvent exposed and forms a water-bridged interaction with Glu-62 and it was therefore assumed to be doubly protonated to reduce the net negative charge of the protein. All Glu and Asp residues were assumed to be negatively charged and all Lys and Arg residues positively charged, whereas the other residues were neutral. This assignment was checked by the PropKa software [42, 43].

All crystal-water molecules were kept in the calculations, except in set 2, for which one water molecule was deleted to avoid steric clashes with the cyano group in ligand **100**. However, after submission of the results, we run additional calculations with set 2, keeping all crystal-water molecules or deleting one (3FT5) or two (2WI7) water molecules by FES before the **101** → **100** perturbation. The protein–ligand complex and the free ligand were solvated in a truncated octahedral box of TIP3P water molecules [79], extending 10 Å from the protein and the ligand, respectively.

The proteins were described with the Amber14SB force field [64] and no counter ions were added to the system. All ligands were manually built into the corresponding protein structure and were described with general Amber force field [80]. Charges were obtained with the restrained electrostatic potential method [81]: the ligands were optimised with the semiempirical AM1 method, followed by a single-point calculation at the Hartree–Fock/6-31G* level to obtain the electrostatic potentials, sampled with the Merz–Kollman scheme [82]. These calculations were performed with the Gaussian 09 software [83]. The potentials were then used by antechamber to calculate the charges. A few missing parameters were obtained with the Seminario approach [84]: the geometry of the ligands was optimised at TPSS/def2-SV(P) level, followed by a frequency calculation using aoforce module of Turbomole 7.01 [60]. From the resulting Hessian matrix, parameters for the missing angles and dihedrals were extracted with the Hess2FF program [85]. These parameters are given in Tables S1 and S2 in the supplementary material.

After submission of the results, it was discovered that the structures of the set 1 ligands were strange, with a tetrahedral –NH_2_ group, accepting hydrogen bonds from the protein and water molecules (Figure S2 in the supplementary material). This was traced back to a missing improper torsion for this group. By adding this torsion with a force constant of 10 kcal/mol/rad2 (cf. Table S2), more reasonable structures were obtained.

In order to estimate the relative binding free energy between two ligands, L_1_ and L_2_, ΔΔ*G*°_bind_ = Δ*G*°_bind_(L_2_) − Δ*G*°_bind_(L_1_), we employed a thermodynamic cycle that relates ΔΔ*G*°_bind_ to the free energy of alchemically transforming L_1_ into L_2_ when they are either bound to the protein, Δ*G*°_bound_, or free in solution, Δ*G*°_free_ [86],

5 $$\Delta \Delta G_{\text{bind}}^{ \circ } = \, \Delta G_{\text{bind}}^{ \circ } \left( {L_{2} } \right) \, {-} \, \Delta G_{\text{bind}}^{ \circ } \left( {L_{1} } \right) \, = \, \Delta G_{\text{bound}}^{ \circ } {-} \, \Delta G_{\text{free}}^{ \circ } .$$

After dividing the transformation of L_1_ to L_2_ into a discrete number of states, described by a coupling parameter λ, multi-state Bennett acceptance-ratio method (MBAR) was used to calculate Δ*G*_bound_ and Δ*G*_free_ [87], using the pyMBAR software [88]. Energies were also calculated with Bennett acceptance ratio (BAR) [89], thermodynamic integration (TI) [90], and exponential averaging (EA) [91]. Separate calculations for the ligand free in water and bound to the protein and 13 intermediate states were used (λ = 0.00, 0.05, 0.10, 0.20, 0.30, 0.40, 0.50, 0.60, 0.70, 0.80, 0.90, 0.95, and 1.00). The electrostatic and van der Waals interactions were perturbed simultaneously in each simulation using soft-core potentials for both types of interactions [92, 93].

For all ligands in set 1 and ligands **10**, **15**, **21**, **23**, **26**, **28**, and **34** in set 3, there are two possible orientations of the modified ring system. No flipping of this ring was observed during the simulations in the protein. Therefore, we run two independent perturbations starting from the two different conformations, in order to enhance the sampling. The resulting dihedral angles in the simulations and the docked structures are shown in Table S3 in the Supplementary material. Ligand **61** in set 3 has two possible configurations (*R* and *S*) and we studied both (experimentally, the racemate was studied [23]).

The alchemical perturbation simulations were performed in the following way [10]: the system at each lambda value was subjected to 100 cycles of steepest-descent minimisation, with all atoms, except water molecules and hydrogen atoms, restrained to their start position with a force constant of 418 kJ/mol/Å^2^. This was followed by 50 ps NPT simulation and a 500 ps NPT equilibration without any restraints. Finally, a 1 ns production simulation was run. Energy differences for MBAR were sampled every 10 ps.

All minimisations and simulations were performed with the pmemd module of Amber14 [54, 94]. The temperature was kept constant at 300 K using a Langevin thermostat with a collision frequency of 2.0 ps^−1^ [95] and the pressure was kept constant at 1 atm using a weak-coupling isotropic algorithm with a relaxation time of 1 ps [96]. Long-range electrostatics were treated by particle-mesh Ewald method [97]. The cutoff for the van der Waals interactions was set to 8 Å. All bonds involving hydrogen atoms were constrained using the SHAKE algorithm [98], so that a time step of 2 fs could be used.

### GCMC calculations

To determine the number of water molecules in the binding site of the set 2 ligand, we employed grand canonical Monte Carlo (GCMC) calculations, as implemented by Essex and coworkers [99] in the ProtoMS software package (version 3.2) [100]. The water structure was analysed for a rectangular box, extending 3 Å in all directions from the ligand, starting from the docked results. The proteins (both 2WI7 and 3FT5) were described with the Amber 14SB force field [64] and the ligands with the general Amber force field [80]. The structures were minimised using AMBER 14 [54] (100 steps minimisation via steepest descent) and then solvated with TIP4P water up to a radius of 10 Å around the protein. All the simulations were performed at 298 K, with a 10 Å cutoff for the non-bonded interactions.

Apart from standard Monte Carlo moves, such as translation and rotation, which apply to the whole system, attempts were also made to insert or delete a water molecule within the box region. The probability is controlled by the chemical potential of an ideal-gas reservoir to which the region around the ligand is being coupled. A virtual titration was performed, simulating the system at different chemical potentials (measured by the Adams value [101]). The optimal number of water molecules around the ligand was determined from the titration curve based on the simulation for which the average number of water molecules corresponds to the binding free energy minimum [99]. The simulation with this value of the chemical potential was analysed to obtain water clusters and these were used as starting positions in FES calculations.

For all systems, GCMC simulations were run for 40 evenly spaced Adams values between −20 and +19. The systems were first equilibrated with 10 million Monte Carlo moves. The first 5 million moves were dedicated to inserting, deleting, and moving water molecules within the box region. In the following 5 million moves, translations and rotations of the protein, the ligand, and the rest of the solvent were introduced for every second move, while the other moves were still dedicated to the water molecules within the box. After the equilibration, we performed 200 million moves of production, where the sampling continued in the same manner. Snapshots were recorded every 0.5 million moves of the production.

### Quality measures and uncertainty estimates

The uncertainties of the free-energy estimates were obtained by nonparametric bootstrap sampling (using 100 samples) of the work values in the MBAR calculations using the pyMBAR software [88]. The other approaches (docking, MM/GBSA, and QM/MM) are based on single structures and therefore do not provide any statistical estimate of the uncertainties. The quality of the binding-affinity estimates compared to experimental data [23] was quantified using the mean absolute deviation (MAD), the squared Pearson’s correlation coefficient (*R* ^2^), and the Kendall’s rank correlation coefficient (τ). The uncertainties of the quality metrics were obtained by a parametric bootstrap (500 samples) using the uncertainties in both the calculated and experimental estimates. The experimental binding affinities were estimated from the measured IC_50_ values [23] according to Δ*G*°_bind_ = *RT* ln(IC_50_/*C*°), where *R* is the ideal gas constant, *T* is the temperature, 300 K, and *C*° is the standard-state concentration, 1 M. Ligand **61** was reported as a non-binder, i.e. having IC_50_ > 50 µM [23] and it was assigned a binding affinity of −24.6 kJ/mol (corresponding to IC_50_ = 50 µM). No uncertainties for the experimental affinities were provided by the organisers. Therefore, we instead assumed a typical uncertainty of 1.7 kJ/mol for the experimental affinities [102] when calculating the uncertainties of the quality measures.

To estimate the convergence of the various perturbations, six different overlap measures were employed [10]. We calculated the Bhattacharyya coefficient for the energy distribution overlap (Ω) [103], the Wu & Kofke overlap measures of the energy probability distributions (*K*_AB_) and their bias metrics (Π) [104, 105], the weight of the maximum term in the exponential average (*w*_max_) [22], the difference of the forward and backward exponential average estimate (ΔΔ*G*_EA_), and the difference between the BAR and TI estimates) [10]. Ω goes from 0, no overlap to 1, perfect overlap [103], and we consider values higher than 0.7 acceptable [10]. *K*_AB_ goes from 0—no overlap, via 1—full overlap, to 2—the first distribution is completely inside the second distribution [104, 105], and again values larger than 0.7 are accepted. A negative Π indicates poor overlap and values below 0.5 are alarming [104, 105]. 1/*w*_max_ indicates how many snapshots contribute significantly to the EA estimate and *w*_max_ values larger than 0.3 indicate poor convergence [10]. ΔΔ*G*_EA_ is the hysteresis in the forward and backward EA estimates, whereas ΔΔ*G*_TI_ indicates the difference between the BAR and TI estimates. In both cases, differences larger than 4 kJ/mol indicate poor convergence [10]. We examined these overlap measures for each of the 26 individual perturbations (13 λ values for simulations with or without the protein). If two of the measures indicated poor overlap (or if Π was negative), additional simulations with intermediate λ values were run.

## Results and discussion

In the present work, we studied three congeneric series of HSP90 inhibitors, shown in Fig. 1, within the D3R 2015 grand challenge blind competition [23]. Sets 1 and 2 are small aminopyrimidine derivatives consisting of five and four molecules, respectively, both containing a 1,3-difluorobenzene group. Set 3 is comprised of ten benzimidazolone derivatives with a 1,3-dihydroxybenzene moiety as the common scaffold. We have estimated absolute binding affinities with molecular docking, MM/GBSA, and QM/MM calculations and relative binding free energies with the FES method. In the following, we will describe the binding modes and affinities obtained with the various methods in separate sections.

### Prediction of binding modes by docking

Initial attempts using a standard docking approach, in which the receptor structure was kept rigid, did not yield satisfactory results, in that only a few ligands docked into the binding pocket. A closer inspection showed that the selected reference crystal structures contain ligands that are smaller than the studied inhibitors, although they contain the proper structural scaffolds. Therefore, steric clashes with either protein residues or surrounding water molecules occurred during the docking of most ligands. To account for protein flexibility, we instead employed the induced-fit docking (IFD) protocol [50, 51], which iteratively performs docking calculations and optimises the protein–ligand complexes through MM minimisations, effectively modelling protein structural changes upon ligand binding. This gave reasonable structures for all complexes.

**Fig. 3:**
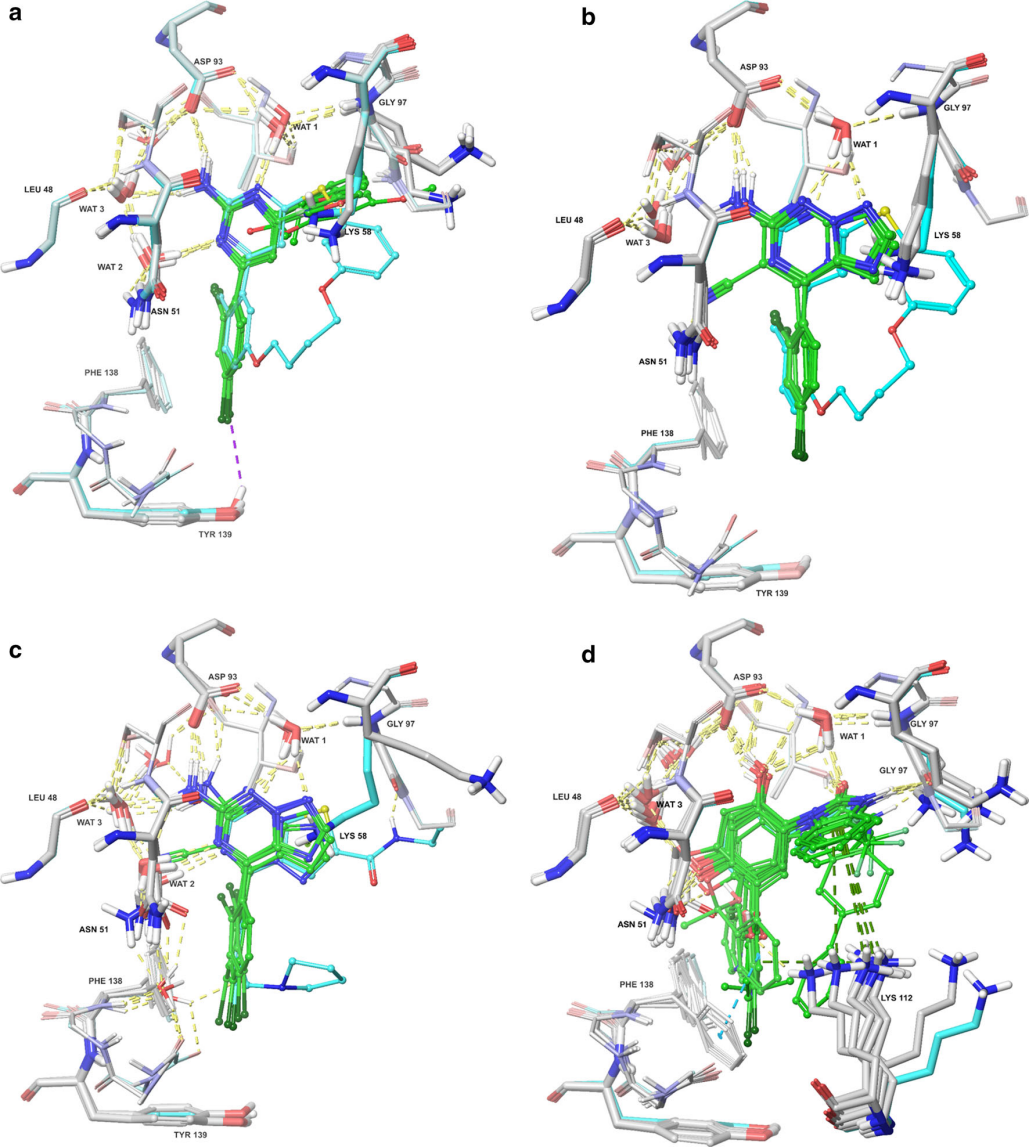
Binding modes for the three series of HSP90 inhibitor from the docking calculations: **a** set 1, **b** original docking for set 2, based on the 3VHA crystal structure (submitted), **c** set 2 in the 2WI7 crystal structure, keeping all water molecules, and **d** set 3. Carbon atoms of the residues are shown in *light grey tubes*, showing some movements as result of the induced-fit docking protocol. Carbon atoms of the ligands are shown as *green tubes*. Water molecules that interact with the ligands are displayed in *thick tube* representation and labelled as WAT. Reference crystal structures (3VHA, 2WI7, and 3OW6 [36, 37, 39]) are coloured in *cyan* for comparison (both ligands and protein). Nitrogen and oxygen atoms are *blue* and *red*, respectively. Hydrogen bonds are represented as *yellow dashed lines* (*purple* if the acceptor is a halogen atom). Cation-π and π-stacking interactions are represented as *dark green* and *dark cyan dashed lines*, respectively

All ligands bound approximately in the same position and orientation as their corresponding reference structure (Fig. 3), displaying favourable interactions with Asp93 and Gly97 in complex hydrogen-bond networks that involve several conserved water molecules. A summary of the protein–ligand interactions is given in Table 2. It shows that all ligands established a strong hydrogen bond with the Asp93 sidechain (H–O distances of 1.96 ± 0.09 Å). Moreover, most of the ligands displayed additional water-bridged hydrogen bonds with Asp93 and Gly97 via one crystal-water molecule (denoted Wat1). Most complexes also showed a stacked interaction between one of the benzene rings and the sidechain of Asn51, with a distance of ~4 Å between the N^ε2^ atom of Asn51 and the centre of the benzene ring [106, 107].

**Table 2:** Hydrogen bonds (first eight lines) and cation–π interactions (last line, Lys122) in the structures obtained with the induced-fit docking

Residues	Set 1		Set 2		Set 2^a^		Set 3	
	*n*	*r*	*n*	*r*	*n*	*r*	*n*	*r*
Lys58	1	2.12						
Asp93	5	2.08 ± 0.09	4	1.86 ± 0.07	4	1.90 ± 0.50	10	1.94 ± 0.11
Wat1	5	1.90 ± 0.07	4	2.12 ± 0.14	4	2.13 ± 0.15	10	1.84 ± 0.09
Wat2	5	1.94 ± 0.07			3	2.10 ± 0.05		
Asn51			1	2.27			8	2.17 ± 0.12
Wat3					4	2.20 ± 0.05	9	1.97 ± 0.17
Gly97							10	2.19 ± 0.14
Thr184							10	1.83 ± 0.08
Asn51^b^	5	4.08 ± 0.05	4	4.30 ± 0.17	4	5.38 ± 0.71	5	4.13 ± 0.71
Lys112							8	5.40 ± 0.76

For each interaction, the number of structures in which this interaction is found is given (*n*, out of 5, 4, and 10 structures for sets 1–3, respectively) and the average distance in these structures (*r* in Å), together with the standard deviation over the *n* structures. Wat1–Wat3 are crystal-water molecules

^a^A second set of docking calculations for set 2, using the 2WI7 crystal structure and keeping Wat2 for ligands **101**, **105**, and **106** (but not **100**), done after the experimental results were revealed

^b^Interaction in which the plane of the sidechain amide group is nearly parallel to the plane of the aromatic ring. The average distance between the N^ε2^ of Asn51 and the centre of the aromatic ring is given

Set 1 ligands also exhibited hydrogen bonds with another crystal-water molecule (Wat2) that directly interacts with Asn51, as well as with Leu48, Ser52, and Thr184 in a network involving two additional water molecules (Fig. 3a). A weak hydrogen-bond with Tyr139 was also identified, where one of the chlorine atom acts as acceptor. Other minor interactions include weak π-stacking interactions with Phe138 and hydrophobic contacts with Lys58. The latter residue showed major variations in the sidechain conformation in the various structures, because this is the only residue that interacts with the variable part of the ligands. In fact, ligand **80** showed a hydrogen-bond with Lys58 sidechain instead, in which the furan oxygen atom acted as the acceptor. For all the other ligands, the sidechain of Lys58 was bent away from the ligand.

The set 2 ligands displayed interactions only with Asp93 and Wat1. However, the cyanide substituent of compound 100 replaced the role of Wat2 in Set 1 and established a hydrogen-bond with Asn51 (cf. Figure 3b). To make the results comparable, Wat2 was excluded in the calculations for all four ligands. After submission, we also tested docking calculations based on the 2WI7 crystal structure (which has a ligand that is chemically more similar to the set 2 scaffold) and kept Wat2 when docking ligands **101**, **105**, and **106**. The results (also included in Table 2) showed that these three ligands can make strong hydrogen bonds to Wat2. Strong interactions with Wat3 and Wat1 were also observed, whereas the interactions with Asp93 became more variable (Fig. 3c). The water molecules bridged interactions with Leu48, Asn51, Asp93, and Gly97. Moreover, the pyrazole ring nitrogen of ligands **105** and **106** established a second hydrogen bond with Wat1 (Fig. 3c).

In general, set 3 inhibitors exhibited a larger number of interactions, and also shorter distances than in the other two sets. In particular, the presence of hydroxyl and carbonyl groups allowed the formation of additional short direct hydrogen bonds with Gly97 and Thr184, where one of the hydroxyl substituents appears to have displaced Wat2 (not present in the reference crystal, 3OW6) in favour of direct hydrogen bonds with Asn51, and allowed for reaching water Wat3, establishing further hydrogen bonds with Leu48, Ser52, Ile91, and Asp93. Major movements were observed for the Lys112 and Phe138 sidechains (Fig. 3d), which were shifted towards the ligands to form cation–π and π-stacking interactions, respectively. The geometry of the cation–π interaction with Lys112 showed a great variability, indicating that this interaction may be important for regulation of the activity. For ligand **61**, only the R conformation was found to bind to the protein in a reasonable mode.

### Binding affinities estimated by docking and MM/GBSA

**Fig. 4:**
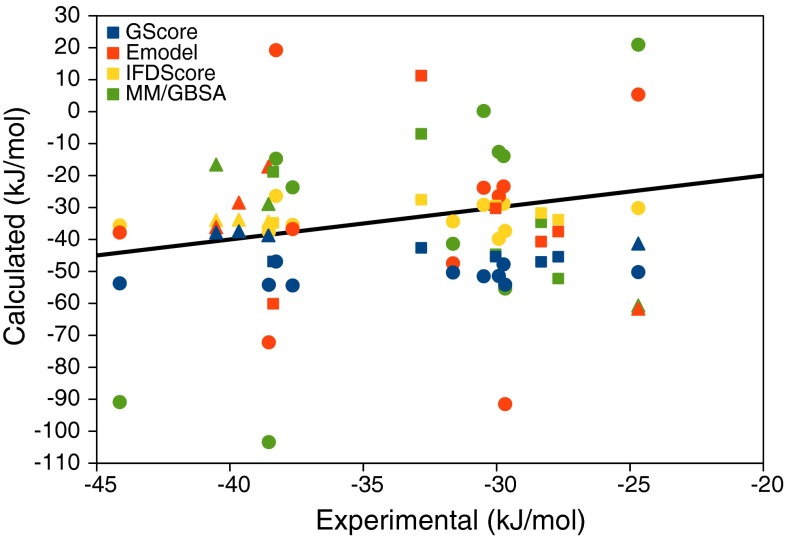
Correlation between the experimental [23] and calculated binding affinities. Sets 1–3 are marked with *squares*, *triangles*, and *circles*, respectively. For GScore, the original score is shown, whereas for E_model_, IFDScore, and MM/GBSA, the mean signed error is subtracted (to give a similar scale of all the calculated results). The *line* shows the perfect correlation. Ligand **61** was experimentally found to be a non-binder, i.e. with a *K*_i_ > 50 µM, which corresponds to ∆*G*_bind_ > −25 kJ/mol

We have estimated the binding affinities for the three sets of ligands with three scoring functions (all employing the same final IFD structures in Fig. 3): GlideScore (GScore), E_model_ and IFDScore (which is the GScore plus a portion of the Prime MM energy from the refinement calculation). In addition, all docked complexes were scored with MM/GBSA calculations, after minimisation of the docked structures. The calculated binding affinities are shown in Table 3. The performance of the tested scores was evaluated by three quality metrics: the correlation coefficient (*R*), Kendall’s rank correction coefficient (τ), and the mean absolute deviation after removal of the systematic error (i.e. the mean signed error; MADtr), which are listed at the bottom of Table 3. The correlation between the experimental [23] and calculated binding affinities are shown in Fig. 4.

**Table 3:** Binding affinities (∆*G*_bind_ in kJ/mol) for the three studied HSP90 inhibitor sets calculated with Glide (GScore and E_model_), induced-fit docking protocol (IFDScore), and MM/GBSA. In addition, the experimental data [23] are included (Exp.)

	Ligand	Exp.	GScore	E_model_	IFDScore	MM/GBSA
Set 1	**80**	−32.6	−42.6	−326.8	−2079.7	−367.9
	**81**	−38.2	−46.9	−398.1	−2086.9	−379.7
	**82**	−28.2	−47.0	−378.7	−2083.8	−395.5
	**83**	−27.5	−45.4	−375.6	−2085.9	−413.1
	**84**	−29.9	−45.4	−368.3	−2081.5	−405.6
Set 2	**100**	−24.6	−41.3	−333.9	−2054.0	−342.8
3VHA	**101**	−38.3	−38.7	−289.5	−2047.0	−311.1
	**105**	−39.5	−37.4	−300.7	−2046.5	−319.5
	**106**	−40.3	−37.8	−308.4	−2046.6	−298.7
Set 2	**100**	−24.6	−39.7	−282.2	−1973.7	−357.2
2WI7	**101**	−38.3	−38.4	−282.9	−1973.4	−310.9
	**105**	−39.5	−40.7	−307.1	−1978.7	−348.0
	**106**	−40.3	−39.5	−309.7	−1974.8	−338.8
Set 3	**10**	−30.3	−51.5	−444.6	−1980.7	−292.2
	**11**	−38.1	−46.9	−401.6	−1977.9	−307.1
	**15**	−29.5	−54.1	−512.3	−1988.8	−347.7
	**19**	−29.6	−47.8	−444.2	−1980.4	−306.3
	**21**	−38.3	−54.2	−493.0	−1989.1	−395.8
	**23**	−31.5	−50.3	−468.3	−1985.9	−333.7
	**26**	−43.9	−53.7	−458.7	−1987.1	−383.3
	**28**	−37.4	−54.4	−457.6	−1986.9	−316.1
	**34**	−29.7	−51.4	−447.2	−1991.3	−305.0
	**61(**R)	>−24.6	−50.2	−415.5	−1981.7	−271.4
MADtr	Set 1		3.9	17.6	3.8	18.2
	Set 2		6.8	18.5	8.3	18.0
	3WI7		5.6	8.7	4.8	17.5
	Set 3		4.3	22.3	5.1	29.4
*R*	Set 1		0.05	0.20	0.21	−0.70
	Set 2		−0.97	−0.86	−1.00	−0.91
	3WI7		−0.02	0.67	0.42	−0.55
	Set 3		0.32	0.11	0.16	0.70
τ	Set 1		−0.20	0.00	−0.20	−0.60
	Set 2		−0.67	0.00	−0.67	−0.67
	3WI7		0.00	1.00	0.33	−0.33
	Set 3		0.20	0.20	0.11	0.42

For set 2, two series of results are given, based on either the 3VHA or 2WI7 crystal structures, the latter including Wat2 for ligands **101**, **105**, and **106**. The lower part of the table contains the quality metrics of the various results: the mean absolute deviation after removal of the systematic error (MADtr), the correlation coefficient (*R*) and Kendall’s rank correlation coefficient (τ). Only the best scores among all obtained structures are reported

The results for set 1 were poor, with a negative or vanishing τ for all methods and a negative (MM/GBSA) or very low correlation (*R* = 0.0–0.2). However, the MADtr is good for both GScore and IFDScore, 4 kJ/mol, but this is mainly an effect of the fact that the range of the predicted affinities is small, 4–7 kJ/mol, compared to experimental range of 11 kJ/mol (setting all calculated affinities to the same value gives a MADtr of 3 kJ/mol).

For the original calculations on set 2, all four methods also gave very poor results, with strong negative correlations (*R* = −0.9 to −1.0), owing to the fact that all methods predicted ligand **100** to bind best, although it experimentally is the weakest ligand. This also gave a large MADtr to all methods (7–18 kJ/mol) and a negative or vanishing τ.

For the calculations based on the 2WI7 crystal structure, in which Wat2 was kept for ligands **101**, **105**, and **106**, the results were more varying (also included in Table 2). The GScore energies showed no correlation with the experimental data, whereas the internal docking score E_model_ produced reasonable correlation (*R* = 0.67) and a correct ligand ranking (τ = 1.00). The IFDScore showed intermediate results (*R* = 0.42 and τ = 0.33). The MM/GBSA results were very poor, with negative *R* and τ. On the other hand, MADtr was best for IFDScore (5 kJ/mol). All methods still predicted ligand **100** to bind with a potency comparable to the other ligands, probably because the employed docking and MM/GBSA rescoring approaches did not consider the cost of displacing Wat2 when ligand **100** binds. In fact, most quality measures improved significantly if ligand **100** was excluded.

For set 3, the results are somewhat better: all methods gave a positive correlation (*R* = 0.1–0.7) and a positive τ (0.1–0.4; however, it should be noted that four of the ligands have experimental affinities within 1 kJ/mol, making it questionable to calculate τ for these—it would be better to consider only statistically significant differences, e.g. τ_90_ [14]). Both *R* and τ were best for MM/GBSA, but MM/GBSA and E_model_ gave poor MADtr (29 and 22 kJ/mol), which reflects that the results for these two methods have a much larger range than the experimental data (124 and 111 compared to 19 kJ/mol). On the other hand, MADtr of GScore and IFDScore is much better, 4 and 5 kJ/mol, but again the ranges are smaller than for the experimental results, 7 and 13 kJ/mol.

Two sets of absolute affinities were submitted, viz. the original GScore and MM/GBSA (submission entries 56afbe93eeaf4 and 56afbea4a8c67, respectively) results in Table 3 (based on the 3VHA structure without Wat2 for set 2).

### QM/MM estimates

Next, we tried to estimate the binding free energies also with a QM/MM approach. As described in the Methods section, we started from the final induced-fit docked structures, to which a sphere of water molecules was added and optimised (together with the hydrogen atoms). Then, a QM system of 280–320 atoms was optimised by QM/MM at the TPSS/def2-SV(P) level of theory (Fig. 2a, b). Finally, a big-QM calculation was performed for a QM system involving all protein residues and water molecules within 7.5 Å of the ligand, 970–1160 atoms, shown in Fig. 2c, d), calculated at the TPSS/def2-SV(P) level of theory in a COSMO continuum solvent. To the big-QM energy, entropy, basis-set, and DFT-D3 dispersion corrections were added, in addition to the relaxation energy and a more accurate COSMO-RS solvation energy of the ligand (Eq. 4).

The QM/MM structures were qualitatively similar to the docked structures, but with some differences in the hydrogen-bond distances, as can be seen by comparing Tables 2 and 4. For set 1, the bonds to Asp93 were shortened, whereas those to Wat1 were elongated. For set 2, the structures of the four ligands were more similar, but the hydrogen-bond interaction with Wat1 was strengthened. For set 3, the hydrogen bonds to Asp93, Wat3, Gly97, and Thr184 were much shortened, whereas that to Wat1 was elongated.

**Table 4:** Hydrogen bonds (first eight lines) and cation–π interactions (last line, Lys122) in the structures obtained with QM/MM optimisation

Residues	Set 1		Set 2		Set 3	
	*n*	*r*	*n*	*r*	*n*	*r*
Lys58	1	2.29				
Asp93	5	1.86 ± 0.04	4	1.83 ± 0.07	10	1.53 ± 0.05
Wat1	5	2.08 ± 0.08	4	1.89 ± 0.05	10	2.07 ± 0.07
Wat2	5	1.87 ± 0.05				
Asn51			1	2.52	9	2.08 ± 0.12
Wat3					9	1.62 ± 0.03
Gly97					10	1.75 ± 0.03
Thr184					10	1.67 ± 0.04
Asn51^a^	5	3.95 ± 0.11	4	4.22 ± 1.03	9	3.75 ± 0.21
Lys112					10	5.42 ± 0.29

For each interaction, the number of structures in which this interaction is found is given (*n*, out of 5, 4, and 10 structures for sets 1–3, respectively) and the average distance in these structures (*r* in Å), together with the standard deviation over the *n* structures. Wat1–Wat3 are crystal-water molecules

^a^Interaction in which the plane of the sidechain amide group is nearly parallel to the plane of the aromatic ring. The average distance between the N^ε2^ of Asn51 and the centre of the aromatic ring is given

Table 5 shows the various QM/MM (free) energy components for the 19 ligands and their correlation to the experimental data. It can be seen that the raw QM/MM energies were large and negative (−620 kJ/mol on average). The same applies to the ${E_{\text{QM1 + ptch2}}^{\text{HL}} }$ energy component (−557 kJ/mol on average), showing that the QM/MM energy is dominated by the QM energy. Neither term showed any convincing correlation to experimental data. The big-QM energies were less negative, especially in the COSMO solvent (−127 kJ/mol on average). However, the correlation to the experimental data was still poor for all three sets of ligands, *R* = −0.1 to 0.3.

**Table 5:** The various QM/MM (free-) energy terms (kJ/mol): the QM/MM energy (∆*E*_QM/MM_), the ${E_{\text{QM1 + ptch2}}^{\text{HL}} }$ energy (∆*E*_QM+ptch_), the big–QM energy (∆*E*_BQ_), calculated either with a point-charge (ptch) model of the surroundings or with COSMO solvation, the dispersion energy, the basis-set correction energy (Eq. 2), the ∆*G*_therm_ ZPE, entropy, and thermal correction, the ligand relaxation energy (∆*E*_L,rlx_), the ligand solvation energy (∆*G*_L,solv_), calculated either at the COSMO (TPSS/def2–SV(P)) or COSMO–RS (BP/TZVP) levels (the ∆∆*G*_L,solv_ term in Eq. (4) is the difference of those two energy terms), and the final QM/MM binding free energy from Eq. (4) (∆*G*_bind_) and the same energy, excluding the ∆*G*_therm_ and ∆*E*_L,rlx_ terms (∆*G*′_bind_). The last nine lines in the table give MADtr, *R* and τ compared to the experimental data [23]

Ligand	∆*E*_QM/MM_	∆*E*_QM+ptch_	∆*E*_BQ_		*∆E*_disp_	∆*E*_bsc_	∆*G*_therm_	∆*E*_L,rlx_	∆*G*_L,solv_	RS	∆*G*_bind_	∆*G*′_bind_
			ptch	COSMO				QZP	COSMO			
**80**	−484.2	−426.7	−156.2	−56.9	−285.5	149.3	93.1	−27.1	−45.9	−54.8	−64.1	−157.2
**81**	−565.5	−491.4	−214.4	−76.2	−324.3	157.4	111.3	−33.8	−55.4	−69.2	−84.3	−195.5
**82**	−487.3	−421.6	−145.2	−45.9	−316.1	145.3	81.4	−21.5	−42.3	−51.5	−104.6	−186.0
**83**	−550.4	−470.1	−218.3	−82.9	−337.8	160.5	99.2	−32.2	−51.3	−60.5	−119.6	−218.8
**84**	−544.9	−471.9	−158.4	−45.0	−340.5	152.6	80.5	−32.7	−57.7	−65.8	−111.6	−192.1
**100**	−487.8	−425.3	−181.7	−65.7	−249.1	148.0	100.1	−16.2	−65.2	−81.9	−33.9	−133.9
**101**	−475.4	−419.0	−164.5	−67.6	−266.1	158.8	61.1	−10.6	−39.3	−48.1	−94.4	−155.5
**105**	−433.5	−379.7	−157.8	−54.3	−238.3	133.1	86.4	−10.2	−49.6	−63.2	−49.3	−135.7
**106**	−438.7	−384.7	−170.7	−70.4	−231.7	130.5	93.7	−10.7	−51.5	−66.1	−52.7	−146.3
**10**	−760.1	−697.2	−447.5	−242.3	−307.2	196.8	123.8	−54.7	−103.8	−141.1	−136.8	−260.6
**11**	−707.8	−645.2	−336.0	−153.8	−324.3	194.7	112.4	−54.8	−89.7	−122.8	−83.0	−195.4
**15**	−851.1	−705.8	−388.7	−182.1	−353.4	256.5	136.8	−48.2	−101.5	−127.4	−68.1	−204.9
**19**	−711.6	−640.0	−386.5	−215.5	−284.3	191.0	106.6	−31.6	−91.4	−123.6	−138.4	−244.9
**21**	−776.1	−684.7	−349.5	−178.5	−359.3	217.3	150.4	−35.9	−92.7	−120.9	−105.9	−256.3
**23**	−748.1	−676.3	−389.8	−185.3	−338.8	186.2	85.8	−47.0	−98.8	−140.3	−163.7	−249.5
**26**	−726.8	−658.0	−349.4	−176.8	−341.0	203.6	116.0	−34.9	−89.9	−123.9	−129.3	−245.3
**28**	−750.0	−685.2	−379.5	−190.6	−325.4	203.3	107.6	−43.9	−95.2	−125.4	−131.0	−238.6
**34**	−749.5	−687.7	−424.5	−235.0	−291.0	196.4	104.5	−52.2	−100.9	−141.0	−132.7	−237.2
**61**	−687.3	−622.1	−282.7	−93.5	−352.0	187.6	123.8	−60.8	−90.3	−121.0	−42.6	−166.5
MADtr	31.9	25.0	29.0	13.3	16.7	6.0	11.5	4.5	5.2	5.1	21.4	14.5
	27.0	24.2	12.1	7.0	15.5	9.4	10.7	7.7	12.5	14.1	17.1	6.1
	31.6	23.7	37.6	30.1	21.0	16.7	13.9	11.1	7.8	9.6	30.0	23.0
*R*	0.33	0.44	0.33	0.29	−0.26	−0.21	−0.70	0.41	0.36	0.57	−0.67	−0.27
	−0.78	−0.73	−0.83	−0.11	−0.22	0.37	0.49	−0.99	−0.81	−0.76	0.53	0.55
	−0.01	0.09	−0.11	0.05	0.21	−0.08	−0.09	−0.53	−0.40	−0.32	0.27	0.38
*τ*											−0.60	−0.20
											0.33	0.33
											0.07	0.33

The dispersion energy was large and negative, showing a smaller variation than the QM energies (−309 kJ/mol on average). It was compensated by the basis-set correction and the ∆*G*_therm_ terms, which both were positive, 177 and 104 kJ/mol on average. Neither term showed any consistent correlation to the experimental data. The relaxation energy of the ligand was 10–61 kJ/mol, largest for the set 3 ligands and smallest for set 2. It showed only a minor variation depending on whether it was calculated with the def2-SV(P) or def2-QZVP basis sets or with or without the COSMO solvation energy (less than 11 kJ/mol). The COSMO-RS solvation energies of the ligand were −48 to −141 kJ/mol, more negative for the set 3 ligands than for the ligands of the other two sets. The COSMO-RS solvation energy was always more negative than the pure COSMO solvation energy, by 23 kJ/mol on average. Neither of the ligand terms showed any consistent correlation to the experimental data.

Adding all the terms according to Eq. 4, we obtained the full QM/MM binding free energy (∆*G*_bind_). From Table 5, it can be seen that it was too negative compared to the experimental data and also with a too large range (−34 to −164 kJ/mol). For sets 2 and 3, it showed a weak correlation with the experimental data (*R* = 0.5 and 0.3, respectively), whereas for set 1, the correlation was negative (*R* = −0.7). For all three sets, MADtr was large, 17–30 kJ/mol. In fact, the results could be improved if the ∆*G*_therm_ and ∆*E*_L,rlx_ terms were omitted (∆*G*’_bind_ column in Table 5). Then, MADtr was only 6 kJ/mol for set 2 and 14–23 kJ/mol for the other two sets. It is often observed with the similar MM/GBSA approach that the results are improved if the ∆*G*_therm_ term is omitted [2]. The reason is probably that the complex and protein structures may relax to different local minima during the MM minimisation. Likewise, MM/GBSA almost invariably exclude the ligand and protein relaxation energies, because they strongly increase the statistical uncertainty of the results [2]. For the rigid octa-acid host–guest system in the SAMPL4 competition, an improvement of the results was obtained if the ligand-relaxation energy was included [16], but with the more flexible ligands in the SAMPL5 competition, the results were deteriorated [108].

Compared to the docking and MM/GBSA results in Table 3, the QM/MM calculations gave much better correlation and τ for set 2, similar or slightly worse results for set 3, and much worse for set 1 (except for MM/GBSA). MADtr was also better for set 2, whereas it was worse than the GScore and IFDScore for the other two sets. One set of relative QM/MM affinities was submitted (submission entry 56af85ab34dbd), viz. the ∆*G*_bind_ results in Table 5, but unfortunately with a sign error in the ∆∆*G*_L,solv_ term in Eq. (4).

### FES results

Relative binding free energies between pairs of ligands were estimated using alchemical FES calculations and employing the standard thermodynamic cycle with the two ligands either bound to the protein or free in solution [86]. Free-energy differences were calculated with the MBAR, BAR, TI, and EA methods. Most of the calculations in sets 1 and 3 involved reference ligands to make the perturbations smaller.

**Fig. 5:**
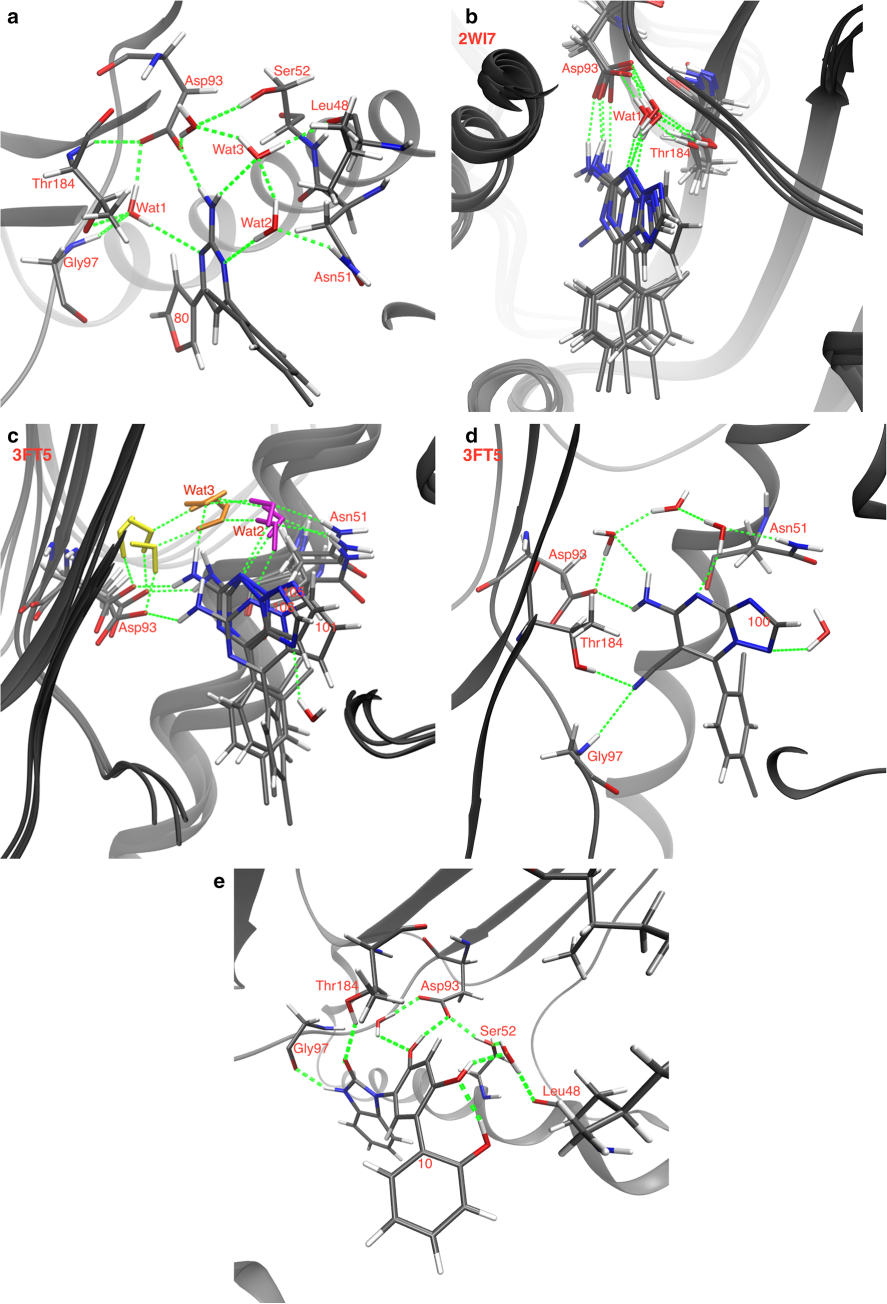
Binding modes in the FES calculations. **a** ligand **80** (set 1; all the other ligands in this set bind in a similar mode), **b** set 2 ligands, based on the 2WI7 crystal structure, **c** ligands **101**, **105**, and **106** (set 2) with three water molecules in different colours (the one in *magenta* corresponds to Wat2 and that in *orange* corresponds to Wat3), **d** ligand **100** (set 2), based on the 3FT5 crystal structure, and **e** ligand **10** (set 3; all the other ligands in this set bind in a similar mode). Hydrogen bonds are indicated by *green dotted lines*

The average structures of the HSP90–ligand complexes are described in Table 6. For set 1, we find that the ligands bind in a mode that is rather similar to that found in the docking and the QM/MM optimisations (Fig. 5a): all ligands formed a direct hydrogen bonds to Asp93 and the two water molecules Wat1 and Wat2, as well as the stacking interaction between the aromatic ring of the ligand and the sidechain of Asn51. However, in variance to the docked and QM/MM structures, all ligands in the FES structures showed also a hydrogen bond to Wat3.

**Table 6:** Hydrogen bonds in the structures obtained in the FES calculations (the most stable conformation of the ligand for Sets 1 and 3)

Residues	Set 1		Set 2 (2WI7)		Set 2 (3FT5)		Set 2 (2WI7 + Wat2)		Set 2 (3FT5 + Wat1)		Set 3	
	*n*	*r*	*n*	*r*	*n*	*r*	*n*	*r*	*n*	*r*	*n*	*r*
Asp93	5	2.01 ± 0.07	4	1.90 ± 0.03	4	2.15 ± 0.10	4	1.87 ± 0.03	3	1.98 ± 0.01	10	1.69 ± 0.04
Wat1	5	2.57 ± 0.15	4	2.16 ± 0.07			4	2.12 ± 0.07			10	2.39 ± 0.09
Wat2	5	2.28 ± 0.09			4	2.17 ± 0.19	3	2.16 ± 0.03	3	2.12 ± 0.11		
Asn51											1	2.11
Wat3	5	2.22 ± 0.04	4	2.33 ± 0.05	1	2.50	4	2.17 ± 0.05	1	2.43	10	1.95 ± 0.27
Gly97					1	2.50					10	2.05 ± 0.05
Thr184					1	2.46					10	1.88 ± 0.09
Asn51^a^	5	3.94 ± 0.06										

For each interaction, the number of structures in which this interaction is found is given (*n*, out of 5, 4, and 10 structures for sets 1–3, respectively) and the average distance for the various ligands over average in the λ = 0 or 1 simulations (*r* in Å), together with the standard deviation over the *n* ligands. Wat1–Wat3 are crystal-water molecules. No cation–π interactions with Lys122 were found for any ligand

^a^Interaction in which the plane of the sidechain amide group is nearly parallel to the plane of the aromatic ring. The average distance between the N^ε2^ of Asn51 and the centre of the aromatic ring is given

Ligands from set 2 bind differently in the FES simulations started from the crystal structures 2WI7 and 3FT5. Structures obtained with the 2WI7 structure were quite similar to the docked and QM/MM structures (Fig. 5b), in which each ligand directly interacted with Asp93 and formed a hydrogen bond network involving Wat1, Asp93, Thr184, and Gly97. No water molecule replaced the deleted Wat2 molecule. In the 3FT5 structures, the ligands still showed a direct hydrogen bond to Asp93, but the ligands were rotated so that the hydrogen-bond network was moved towards Asn51 and involved Wat2, Wat3, and a third water molecule, shown in Fig. 5c. Ligand **100** of the 3ft5 subset also formed two direct hydrogen bonds with Thr184 and Gly97 (Fig. 5d).

**Fig. 6:**
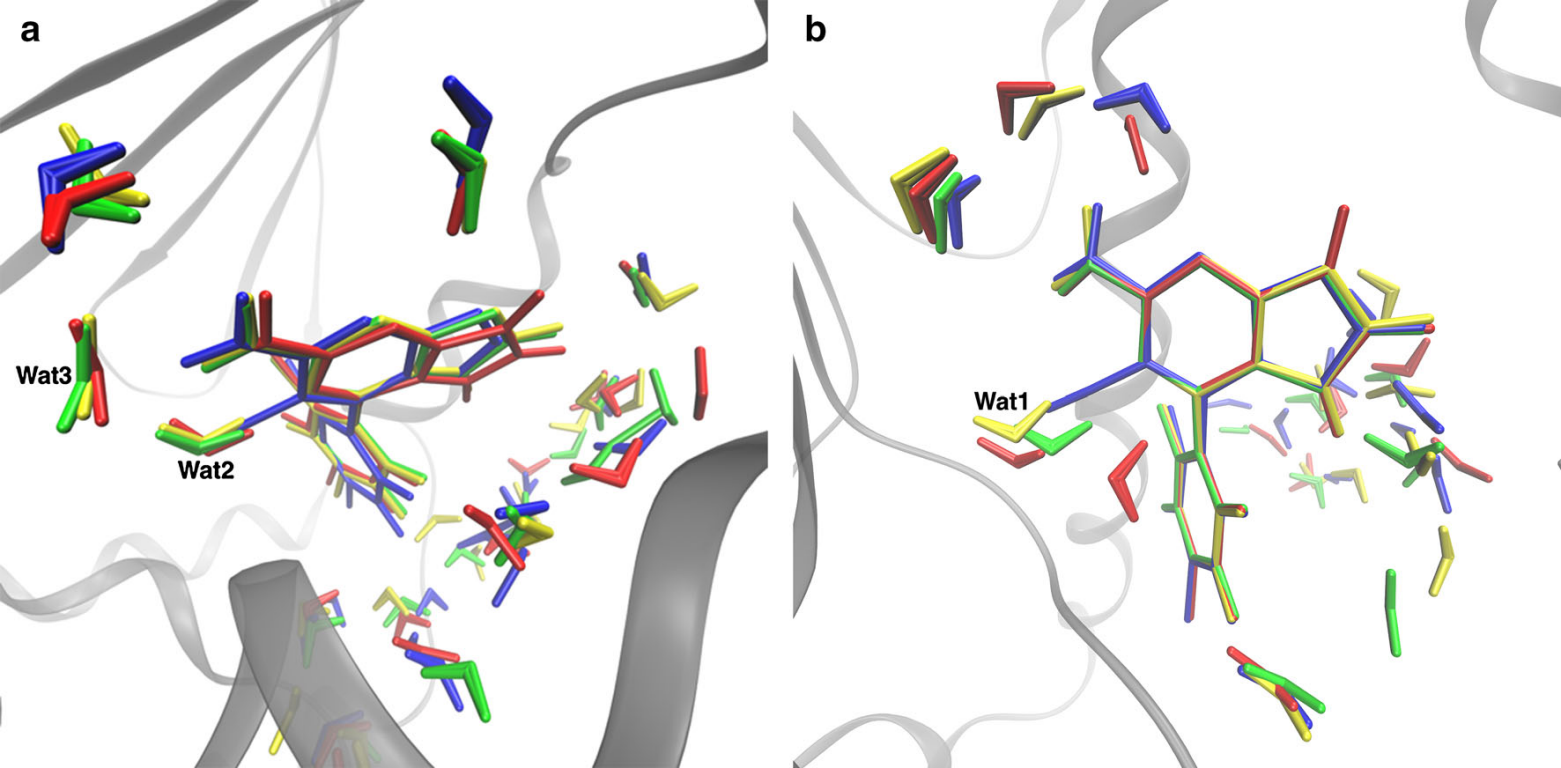
Water clusters obtained by GCMC method for the **a** 2WI7 and **b** 3FT5 structures with set 2 ligands. In both figures, ligands and the corresponding water molecules are presented in different colours: ligand **100**—*blue*, ligand **101**—*red*, ligand **105**—*yellow*, and ligand **106**—*green*

After submission of the results, we performed GCMC calculations to study the water structure around the ligands of set 2. These calculations are described in the Supplementary material. The resulting clustered water molecules around the various ligands are shown in Fig. 6. It can be seen that for the 2WI7 structure, the cyano group in ligand **100** replaced two water molecules that were present for the other three ligands (Wat2 and Wat3). For the 3FT5 structure, only one water molecule (Wat1) was displaced by the cyano group in ligand **100**. Therefore, we performed an additional set of FES calculations (using both the 2WI7 and 3FT5 structures), in which all water molecules were included in the perturbations. For ligand **100** in the 2WI7 structure, Wat2 moved away from the ligand and ended up in bulk solvent, whereas for the other ligands, Wat2 stayed in the original position. Wat3 remained in the starting position in all calculations with the 2WI7 structure (i.e. also for ligand **100**). For the calculations in the 3FT5 structure, Wat1 did not interact directly with any of the ligands (the distance was ~2.7 Å). For ligand **100**, Wat3 came in and bridged the interaction with Asp93. Thereby, it interacted very weakly with the protein.

All the ligands from set 3 bound to the protein in a similar way, with rather small variations between the different ligands. Each ligand forms direct hydrogen bonds with Gly97, Thr184, and Asp93, and also an additional water-bridged interaction with the latter residue. Each ligand also binds to Ser52 and Leu48 via a water molecule (Fig. 5e). These binding modes are quite similar to the ones observed in the docking and the QM/MM results for the set 3. However, we do not find any interaction with Ile91, and Lys112 is far away from the ligand.

For set 1, the perturbations involved mainly the substituents of one of the three ring systems, involving the perturbation of one (or in one case two) hydrogen atoms to methyl, methoxy, or ethoxy groups. In one case, the benzene ring was instead perturbed to a furan ring (ref → **80**). In another case, a methyl group is perturbed to an acetate group (**81** → **82**). Set 2 involves perturbations of C and N atoms in a fused six and five-ring system. In one case (**100**), a cyano group is also added. Set 3 is more diverse, although all ligands share a benzimidazolone group joined to a resorcinol group. By the use of three reference ligands, the size of the perturbations was in many cases reduced to the conversion of hydrogen atoms to hydroxyl, chloride, methoxy, CF_3_, and isopropyl groups, or to the conversion of a carbon atom in the benzene ring to a nitrogen atom (pyridine). However, in one case a hydrogen atom is converted to a benzene ring (**19** → ref1), in one case the benzene ring is converted to quinoline (**23** → ref2), and in one case, the benzene and resorcinol rings are joined by a pyran ring (**61** → ref2).

The raw binding affinities calculated with FES are given in Table 7. It can be seen that the precision of the FES results was reasonable: the standard errors of the MBAR estimates were 0.2–0.9 kJ/mol, indicating good convergence of the perturbations. Results obtained with the BAR, TI, and EA methods are shown in Table S4 in the Supplementary material. The BAR and TI results agreed with the MBAR results with MADs of 0.6 and 0.8 kJ/mol, respectively, which indicates a somewhat worse convergence. In particular, the **21** → ref3 and **26** → ref2 perturbations gave alarming differences of 4 and 5 kJ/mol, respectively. The convergence of all perturbations was examined by considering a set of six overlap measures, as described in the Methods section. All 26 individual simulations for each perturbation were checked for poor overlap and additional simulations were run with intermediate λ values if two of the overlap measures indicated poor overlap or if Π (which is considered to be the most reliable overlap measure, with the best correlation to the other measures [10]) was negative. Consequently, the presented results should be numerically reliable.

**Table 7:** Calculated relative binding free-energies and standard errors (obtained with MBAR in kJ/mol) for the studied perturbations

Transformation	Exp.	Results 1	Results 2	Results 3
Set 1		Conf. 1	Conf. 2	
ref → **80**		1.8 ± 0.5	**−3.6** **±** **0.5**	
**81** → **82**	10.0	**−13.2** **±** **0.5**	−16.4 ± 0.5	
**82** → ref		13.3 ± 0.3	**16.4** **±** **0.3**	
**83** → ref		**3.6** **±** **0.5**	−3.5 ± 0.5	
**84** → ref		**8.3** **±** **0.5**	**8.3** ± 0.6	

Set 2 without Wat1/2		2WI7	3FT5	2WI7/3FT5
**101** → **100**	13.9	−12.2 ± 0.5	2.7 ± 0.5	−12.8 ± 0.5
**101** → **105**	−0.1	−7.5 ± 0.2	2.7 ± 0.2	−8.4 ± 0.2
**101** → **106**	2.0	−7.3 ± 0.3	3.8 ± 0.3	−8.7 ± 0.3

Set 2 with Wat1/2		2WI7	3FT5	
**101** → **100**	13.9	11.2 ± 0.9	18.0 ± 0.9	
**101** → **105**	−0.1	−6.2 ± 0.4	3.5 ± 0.4	
**101** → **106**	2.0	−3.7 ± 0.5	5.5 ± 0.5	

Set 3		Conf. 1	Conf. 2	
**10** → ref2		−4.9 ± 0.4	**−0.6** **±** **0.4**	
**11** → ref2		**2.3** **±** **0.2**		
**15** → ref3		**4.8** **±** **0.5**	−4.1 ± 0.6	
**19** → ref1		**2.9** **±** **0.6**		
**21** → ref3		**7.3** **±** **0.4**	−2.1 ± 0.4	
**23** → ref2		**−6.7** **±** **0.5**	−13.1 ± 0.6	
**26** → ref2		**3.7** **±** **0.4**	−12.0 ± 0.4	
**28** → ref2		**1.3** **±** **0.4**	−1.9 ± 0.4	
**34** → ref2		**−0.2** **±** **0.7**	−3.7 ± 0.7	
**61S** → ref2		**−4.8** **±** **0.8**		
**61R** → ref2		−19.8 ± 0.4		
ref 2 → ref1		**−11.5** **±** **0.6**		
ref 3 → ref2		**4.5** **±** **0.4**		

Experimental data [23] for the relative energies are also given for the transformations that do not involve any reference ligands

As mentioned in the Methods section, many of the ligands in sets 1 and 3 can bind with two conformations, differing by an 180° rotation of the perturbed ring. In the FES calculations, both conformations were tested, starting from the symmetric reference molecules. The best conformation was then selected as the one that gave the most favourable binding energy, compared to the reference molecule (shown in bold face in Table 7). The average dihedral angles observed during the FES simulations and in the docked structures are shown in Table S3. In most structures, the ring systems were not coplanar.

Ligand **61** has two stereoisomers, depending on the orientation of the hydroxyl and methyl groups. We tested both and found the S form to bind more favourably than the R form. This is in striking contrast to the docking calculations, which indicated that only the R form bound to the protein. Experimentally, ligand **61** (racemic mixture) was found to be a non-binder.

For set 2, no reference ligands were employed and therefore, we can directly compare the results of the three studied perturbations with experimental relative affinities. From the results in Table 7, it can be seen that the two results employing the pose in the 2WI7 crystal structure, but using either the 2WI7 or the 3FT5 crystal structures gave similar relative affinities. Therefore, only one of these results is compared with experiments in Table 8. It can be seen that the results were poor with a strongly negative correlation (*R* = −0.8), an incorrect sign for two of the perturbations (*τ*_r_ = −0.3, although the sign of one of the experimental relative affinities in not statistically significant), and a MAD of 14 kJ/mol. However, the results based on the 3FT5 crystal structure were much better with a positive correlation (*R* = 0.6), a correct sign of two of the perturbations (those that have statistically significant experimental differences) and a MAD of 5 kJ/mol. The results of the docking and MM/GBSA calculations (for the same *relative* affinities, also shown in Table 8) were much worse with *R* = −0.6 and −0.8, *τ*_r_ = −1.0, and MAD = 8 and 28 kJ/mol, respectively. QM/MM results were of intermediate quality with *R* = 0.4 and MAD = 13 kJ/mol.

**Table 8:** Performance of the various methods to calculate relative binding free energies (MAD and maximum error, Max, in kJ/mol) compared to experimental results [23]

	GScore	MM/GBSA	QM/MM	FES	
*Set 1*					
MAD	5.8–6.1	20.8–26.3	17.6–29.1	10.9–15.9	
*R*	−0.58 to 0.03	−0.69 to −0.60	−0.42 to −0.01	−0.80 to −0.54	
τ	−1.00 to −0.40	−0.43 to 0.00	−0.14 to 0.50	−1.00 to −0.71	
Max	10.2	32.2–50.4	33.3–66.8	23.3	

				2WI7	3FT5
*Set 2 without Wat1/2*					
MAD	7.7	27.8	12.9	14.2 ± 1.0	5.3 ± 0.8
*R*	−0.57	−0.81	0.43	−0.81 ± 0.07	0.59 ± 0.10
τ	−1.00	−1.00	−0.33	−0.33 ± 0.33	0.33 ± 0.48
Max	16.4	45.6	19.9	26.0 ± 1.8	11.2 ± 1.8

				2WI7	3FT5
*Set 2 with Wat1/2*					
MAD	6.1	41.0		4.8 ± 1.3	3.7 ± 1.3
*R*	0.49	−0.58		1.00 ± 0.04	1.00 ± 0.04
τ	0.33	0.33		0.33 ± 0.43	0.33 ± 0.43
Max	15.2	60.2		6.1 ± 1.8	4.1 ± 1.8
*Set 3*					
MAD	4.7–10.4	29.6–56.0	23.6–47.1	8.7–14.6	
*R*	−0.45 to 0.70	0.18 to 0.92	−0.32 to 0.57	−0.47 to −0.20	
τ	−0.56 to 0.33	0.33 to 0.78	−0.33 to 0.56	−0.78 to 0.11	
Max	8.8–16.9	55.4–95.6	59.1–88.4	17.9–27.9	

For set 1, the reported values are the range obtained when doing three comparisons: four relative affinities using ligand **82** as the reference, all seven relative affinities that can be obtained by combining two perturbations, or all ten possible relative affinities of the five ligands. For set 2, we present the results of the three perturbations studied by FES, reporting bootstrapped uncertainties, using the observed standard error for FES. Values in brackets for GScore and MM/GBSA were obtained using the 2WI7 crystal structure. For set 3, we present the range obtained by using either ligands **10**, **11**, **23**, **26**, **28**, or **34** as the reference

Keeping the Wat2 crystal water molecule in the FES calculations improved the results for both crystal structures, giving a perfect correlation (*R* = 1.0) and low MADs (5 kJ/mol for 2WI7 and 4 kJ/mol for 3FT5). In particular, both sets of calculations predicted that ligand **100** has a much lower binding affinity (~10 kJ/mol) than the other three ligands. However, in both cases, one of the three relative affinities had an incorrect sign (τ_r_ = 0.3), although for the 3FT5 structure this involved the transformation for which the experimental estimate is not statistically significant. These calculations also gave an ideal slope of 1.0, whereas it was 1.2 for the calculations based on the 2WI7 structure. Both FES calculations gave better results than the docking and MM/GBSA calculations including Wat2 (*R* = 0.5 and MAD = 6 kJ/mol for GScore).

For the other two sets of ligands, no direct comparison with experiments [23] can be performed, because all studied perturbations (except one) involved reference ligands with unknown experimental affinities. This means that the calculated results need to be combined to compare with experiments, increasing the uncertainty and making the comparison dependent on which data are combined. Moreover, when calculating the correlation coefficient, the results also depend on the sign of the transformation (i.e. whether the **81** → **82** or **82** → **81** perturbation is considered, for example). The latter problem was solved by always considering both directions of the perturbation when *R* was calculated.

For set 1, it may seem natural to compare with ligand **82**, because all relative affinities can be obtained from this ligand using one or two perturbations. However, three additional relative affinities can be obtained by combining two perturbations and all ten possible relative affinities can be obtained from three perturbations. Therefore, we give in Table 8 the results of three different comparisons (as ranges): four relative affinities using ligand **82** as the reference, all seven relative affinities that can be obtained by combining two perturbations, and all ten possible relative affinities. Numerically, the results vary somewhat, but all results were poor: the correlation was negative (*R* = −0.8 to −0.5), MAD = 11–16 kJ/mol, and *τ*_r_ = −1.0 to −0.7, i.e. only one relative affinity had the correct sign, but the signs of four of the measured relative affinities are not statistically significant. In fact, the largest error (23 kJ/mol) is obtained for the **81** → **82** transformation that is directly comparable with experiments.

The docking gave a smaller MAD and MM/GBSA and QM/MM larger MADs than FES (6, 21–26, and 18–29 kJ/mol, respectively), owing to a smaller and larger ranges of the absolute affinities compared to experiments, 4, 45, and 62 kJ/mol, respectively, compared to 11 kJ/mol for the experimental data. All three methods showed no or negative correlations (*R* = −0.0 to −0.7). Likewise, τ_r_ was mostly negative (−0.1 to −1.0) or zero, except when using ligand **82** as the reference for QM/MM (τ_r_ = 0.5).

For set 3, the situation is even more complicated: all studied transformations involve at least one of the three reference molecules. Any of ligands **10**, **11**, **23**, **26**, **28**, **34**, and **61** can be individually compared employing two perturbations, whereas ligands **19**, **15**, and **21** require the combination of three perturbations. Table 8 shows the range of results obtained when using any of the six ligands in the first group as the reference (excluding ligand 61, because it is experimentally a non-binder). It can be seen that the FES results were quite poor with a negative correlation (*R* = −0.5 to −0.2), a varying τ_r_ (−0.8 to +0.1), a MAD of 9–15 kJ/mol and maximum errors of 18–28 kJ/mol.

From Table 8, it can also be seen that the docked results for set 3 were somewhat better with a positive correlation (*R* = 0.3–0.7), except when ligand **11** was used as the reference (*R* = −0.5). The same applies to τ_r_, which was positive (0.1–0.3), except when using ligand **11** as the reference (τ_r_ = −0.6). MAD was appreciably better 5–10 kJ/mol, but this is mainly because all relative energies were underestimated: the range of the affinities was only 7 kJ/mol, whereas the experimental range was at least 19 kJ/mol, and in FEP the range was 21 kJ/mol. The MM/GBSA calculations vastly overestimated the range (124 kJ/mol) and therefore gave a very poor MAD of 30–56 kJ/mol and a maximum error of up to 124 kJ/mol (9–17 kJ/mol for the docking). On the other hand, the correlation was always positive, reaching an impressive *R* = 0.9 when using ligand **26** as the reference. Likewise, τ_r_ was better than for the other methods, 0.3–0.8. QM/MM gave quite poor results with both *R* and τ_r_ = −0.3 to 0.6 and MAD = 24–47 kJ/mol.

One set of relative affinities was submitted (submission entry 56af858f31db8). It was based on the data in Table 7 for sets 2 (2WI7 structure) and 3, but the data in Table S5 for set 1 (i.e. obtained without the improper ca–hn–nh–hn dihedral angle, giving spurious structures, as discussed above). The data were submitted with ligands **80**, **100**, and **10** as the reference, which increases the uncertainty and may affect the calculated quality estimates. Unfortunately, we selected to submit the set 2 results based on the 2WI7 structure (mainly because the 2WI7/3FT5 results were similar), although it turned out that the 3FT5 reproduced the experimental measurements much better.

## Conclusions

In this study, we have tried to estimate the binding affinities of three sets of ligands (with five, four and ten ligands in each) for HSP90 in the D3R 2015 grand challenge blind-test competition. We have employed four different theoretical methods of varying sophistication: docking with the induced-fit protocol in Glide, MM/GBSA calculations with single minimised structures performed by Prime, a new QM/MM approach, based big-QM calculations with various energy terms added, and standard FES calculations of relative binding affinities.

Unfortunately, the results were quite disappointing, with poor and often negative correlation and τ values for most of the methods and ligand sets. For set 2, the problem could be traced to the displacement of one or two water molecules by one of the ligands. If this effect was properly accounted for, FES and some docking scores gave good results. We employed GCMC calculations to deduce which water molecules dissociate with the various ligands.

Owing to the poor overall results, it is hard to compare the four methods employed. However, our results show no clear-cut advantage of using the more rigorous method FES approach, which comes with a much higher computational effort. In general, the docking calculations with GScore and IFDScore gave small MADtr for all three sets, 4–8 kJ/mol. However, this primarily reflects that these scores underestimate the differences between the various ligands. The E_model_ score and MM/GBSA gave much higher MADtr (9–29 kJ/mol) and a strong overestimation of the range of the calculated binding affinities.

Compared to the other submissions in this blind-test competition, our calculations gave in general mediocre or poor results [23]. However, QM/MM was one of the few methods that gave a non-negative τ and a positive correlation for set 2, and without the unfortunate sign error, the correct QM/MM results would have given the best *R* and τ among all submissions. For set 3, our docked results gave the lowest RMSD and MM/GBSA gave the best τ among all submissions (in fact, our four submissions gave among the five best τ values for set 3). Still, this mainly reflects the large variation in the performance of the results from both us and the other groups; the other submissions also gave rather disappointing overall results: in particular, none of the submissions gave positive τ values for all three sets.

In the new QM/MM method, we first reoptimised the docked structures with standard QM/MM calculations, using a quite large QM system (280–320 atoms), including all atoms within 3 Å of the ligand. Then, the QM system was enlarged with all atoms within 7.5 Å of the ligand (970–1160 atoms) and a single-point energy was calculated in a COSMO continuum solvent (Fig. 2). To the rigid interaction energies calculated with this model, we added five energy corrections (Eq. 4), similar to what has been used for host–guest systems [16, 33, 34]: first, a correction term for increasing basis set for the smaller QM system to quadruple-zeta quality. Second, a DFT-D3 dispersion correction, including third-order terms. Third, a thermostatistical correction, including the zero-point energy and entropy, calculated at the MM level with a free-rotor approximation for the low-lying vibrations. Fourth, a ligand-relaxation energy term, and finally an improved solvation energy for the ligand, estimated by the COSMO-RS approach. We also tried to include the solvation free energy of the whole protein with PB or GB methods, but could not obtain any consistent results.

Unfortunately, the QM/MM affinities, showed no consistent improvement over the docked results, although most hydrogen bonds were shortened. Instead, the QM/MM energies showed a similar overestimation of the differences in the binding affinities as the MM/GBSA method, giving MADtr of 17–30 kJ/mol. Still, the results could consistently be improved for all three sets if the ligand-relaxation and thermostatistical terms are omitted (e.g. MADtr = 6–23 kJ/mol). It is probably necessary to employ more than a single minimised structure to obtain consistent and reliable results with QM/MM.

Clearly, the FES results were disappointing, with MADs of 4–15 kJ/mol and maximum errors of up to 26 kJ/mol. Previous large-scale tests of relative FES affinities have shown that MADs of 2–6 kJ/mol are typically obtained for well-behaving systems [9–11]. Such results were only obtained for set 2 if all water molecules are included. The much larger errors obtained for the other two sets can have several causes. First, some of the perturbations in this study are larger than in the large-scale tests. However, we have thoroughly monitored the overlap, convergence, and precision of the calculations, and there is not indication that the perturbations are too large or that the sampling is too short. On the other hand, HSP90 has a flexible binding site and the simulations are much too short to sample larger conformational changes in the binding site or the whole protein. Second, it is possible that the MM force field is not accurate enough to model the chemical variation of the ligands. However, the set 1 ligands show a rather restricted variation, involving mainly methyl, methoxy, ethoxy, and acetate groups, for which the general Amber force field is expected to perform well.

Third, for all FES calculations, we have assumed that all ligands bind in the same mode as the starting crystal structure. Some differences have been observed between the FES and docked structures and also between the various starting structures. If the binding mode in the crystal structure is incorrect or if the binding mode changes between the various ligands, FES is expected to give poor results, and this would affect also the other calculations, because docked structures were accepted only if they were similar to the crystal structures. We believe that this is the main reason for the poor results in this investigation. It should also be noted that the variable parts of the ligands do not show much interactions with the protein. This means that there is a risk that the ligands may bind in a different conformation and that some residues in the protein may show a large change in conformation (to form interactions with this part of the ligand), or that the binding is mainly determined by the interaction of this part with solvent. Clearly, all ranking methods heavily depend on accurate structures, but unfortunately, crystal structures are lacking for all ligands in this investigation. This makes the present test somewhat less informative when it comes to the ranking of different methods to predict binding affinities. To obtain improved binding-affinity predictions for such complicated systems, FES methods involving enhanced sampling could be tested, e.g. metadynamics, accelerated MD, or replica-exchange methods [109–114]. However, many of them are most effective if it is known beforehand which groups need better sampling, which not always is the case. They also significantly increase the computational effort.

## Electronic supplementary material

Below is the link to the electronic supplementary material.

Supplementary material 1 (PDF 598 kb)

## References

[1] Gohlke H, Klebe G (2002). Angew Chem Int Ed.

[2] Genheden S, Ryde U (2015). Expert Opin Drug Discov.

[3] Åqvist L, Luzhkov VB, Brandsdal BO (2002). Acc Chem Res.

[4] Mobley DL, Klimovich PV (2012). J Chem Phys.

[5] Warren GL, Andrews CW, Capelli AM, Clarke B, LaLonde J, Lambert MH, Lindvall M, Nevins N, Semus SF, Senger S (2006). J Med Chem.

[6] Cross JB, Thompson DC, Rai BK, Baber JC, Fan KY, Hu Y, Humblet C (2009). J Chem Inf Model.

[7] Brown SP, Muchmore SW (2009). J Med Chem.

[8] Sun H, Li Y, Tian S, Xu L, Hou T (2014). Phys Chem Chem Phys.

[9] Christ C, Fox T (2014). J Chem Inf Model.

[10] Mikulskis P, Genheden S, Ryde U (2014). J Chem Inf Model.

[11] Wang L, Wu Y, Deng Y, Kim B, Pierce L, Krilov G, Lupyan D, Robinson S, Dahlgren MK, Greenwood J, Romero DL, Masse C, Knight JL, Steinbrecher T, Beuming T, Damm W, Harder E, Sherman W, Brewer M, Wester R, Murcko M, Frye L, Farid R, Ling T, Mobley DL, Jorgensen WL, Berner BJ, Friesner RA, Abel R (2015). J Am Chem Soc.

[12] Genheden S, Luchko T, Gusarov S, Kovalenko A, Ryde U (2010). J Phys Chem B.

[13] Genheden S, Nilsson I, Ryde U (2011). J Chem Inf Model.

[14] Mikulskis P, Genheden S, Rydberg P, Sandberg L, Olsen L, Ryde U (2012). J Comput Aided Mol Des.

[15] Muddana HS, Fenley AT, Mobley DL, Gilson MK (2014). J Comput Aided Mol Des.

[16] Mikulskis P, Cioloboc D, Andrejic M, Khare S, Brorsson J, Genheden S, Mata RA, Söderhjelm P, Ryde U (2014). J Comput Aided Mol Des.

[17] Coleman RG, Sterling T, Weiss DR (2014). J Comput Aided Mol Des.

[18] Naïm M, Bhat S, Rankin KN, Dennis S, Chowdhury SF, Siddiqi I, Drabik P, Sulea T, Bayly CI, Jakalian A, Purisima EO (2007). J Chem Inf Model.

[19] Muddana HS, Varnado CD, Bielawski CW, Urbach AR, Isaacs L, Geballe MT, Gilson MK (2012). J Comput Aided Mol Des.

[20] Mobley DL, Liu S, Lim NM, Wymer KL, Perryman AL, Forli S, Deng N, Su J, Branson K, Olson AJ (2014). J Comput Aided Mol Des.

[21] Hogues H, Sulea T, Purisima EO (2014). J Comput Aided Mol Des.

[22] Gallicchio E, Deng N, He P, Perryman AL, Santiago DN, Forli S, Olson AJ, Levy RM (2014). J Comput Aided Mol Des.

[23] Gathiaka S, Liu S, Chiu M, Yang H, Burley SK, Walters WP, Amaro RE, Gilson MK, Feher VA (2016) D3R grand challenge 2015: evaluation of protein–ligand pose and affinity predictions. J Comput Aided Mol Des. doi:10.1007/s10822-016-9946-8 10.1007/s10822-016-9946-8 PMC5562487 27696240

[24] Lindquist S, Craig EA (1988). Annu Rev Genet.

[25] Isaacs JS, Xu W, Neckers L (2003). Cancer Cell.

[26] Cullinan SB, Whitesell L (2006). Semin Oncol.

[27] Solit DB, Rosen N (2006). Curr Top Med Chem.

[28] McDonald E, Workman P, Jones K (2006). Curr Top Med Chem.

[29] Huth JR, Park C, Petros AM, Kunzer AR, Wendt MD, Wang X, Lynch CL, Mack JC, Swift KM, Judge RA, Chen J, Richardson PL, Jin S, Tahir SK, Matayoshi ED, Dorwin SA, Ladror US, Severin JM, Walter KA, Bartley DM, Fesik SW, Elmore SW, Hajduk PJ (2007). Chem Biol Drug Des.

[30] Brunko M, Tahir SK, Song X, Chen J, Ding H, Huth JR, Judge RA, Madar DJ, Park CH, Park CM, Petros AM, Tse C, Rosenberg SH, Elmore SW (2010). Bioorg Med Chem Lett.

[31] Glide, version 6.7, Schrödinger, LLC, New York, NY, 2015

[32] Prime, version 4.0, Schrödinger, LLC, New York, NY, 2015

[33] Grimme S (2012). Chem Eur J.

[34] Antony J, Sure R, Grimme S (2015). Chem Commun.

[35] Hu L, Söderhjelm P, Ryde U (2013). J Chem Theory Comput.

[36] Bruncko M, Tahir SK, Song X (2010). Bioorganic Med Chem Lett.

[37] Brough PA, Barril X, Borgognoni J (2009). J Med Chem.

[38] Barker JJ, Barker O, Boggio R, Chauhan V, Cheng RK, Corden V, Courtney SM, Edwards N, Falque VM, Fusar F, Gardiner M, Hamelin EM, Hesterkamp T, Ichihara O, Jones RS, Mather O, Mercurio C, Minucci S, Montalbetti CA, Muller A, Patel D, Phillips BG, Varasi M, Whittaker M, Winkler D, Yarnold CJ (2009). Chem Med Chem.

[39] Suda A, Koyano H, Hayase T (2012). Bioorganic Med Chem Lett.

[40] Kang YN, Stuckey JA Structure of Heat Shock Protein 90 Bound to CS302. To be published, PDB structure 4YKR

[41] Schrödinger Suite 2015-2 Protein Preparation Wizard; Epik version 3.2, Schrödinger, LLC, New York, NY, 2015; Impact version 6.7, Schrödinger, LLC, New York, NY, 2015; Prime version 4.0, Schrödinger, LLC, New York, NY, 2015

[42] Olsson MHM, Søndergaard CR, Rostkowski M, Jensen JH (2011). J Chem Theory Comput.

[43] Søndergaard CR, Olsson MHM, Rostkowski M, Jensen JH (2011). J Chem Theory Comput.

[44] Sastry GM, Adzhigirey M, Day T (2013). J Comput Aided Mol Des.

[45] Banks JL, Beard HS, Cao Y, Cho AE, Damm W, Farid R, Felts AK, Halgren TA, Mainz DT, Maple JR, Murphy R, Philipp DM, Repasky MP, Zhang LY, Berne BJ, Friesner RA, Gallicchio E, Levy RM (2005). J Comp Chem.

[46] Maestro, version 10.2, Schrödinger, LLC, New York, NY, 2015

[47] LigPrep, version 3.4, Schrödinger, LLC, New York, NY, 2015

[48] Epik, version 3.2, Schrödinger, LLC, New York, NY, 2015

[49] MacroModel, version 10.8, Schrödinger, LLC, New York, NY, 2015

[50] Sherman W, Day T, Jacobson MP (2006). J Med Chem.

[51] Schrödinger Suite 2015-2 Induced Fit Docking protocol; Glide version 6.7, Schrödinger, LLC, New York, NY, 2015; Prime version 4.0, Schrödinger, LLC, New York, NY, 2015

[52] Li J, Abel R, Zhu K (2011). Proteins Struct Funct Genet.

[53] Mulakala C, Viswanadhan VN (2013). J Mol Graph Model.

[54] Case DA, Berryman JT, Betz RM, Cerutti DS, Cheatham TE, Darden TA, Duke RE, Giese TJ, Gohlke H, Goetz AW, Homeyer N, Izadi S, Janowski P, Kaus J, Kovalenko A, Lee TS, LeGrand S, Li P, Luchko T, Luo R, Madej B, Merz KM, Monard G, Needham P, Nguyen H, Nguyen HT, Omelyan I, Onufriev A, Roe DR, Roitberg A, Salomon-Ferrer R, Simmerling CL, Smith W, Swails J, Walker RC, Wang J, Wolf RM, Wu X, York DM, Kollman PA (2014). AMBER 14.

[55] Ryde U (1996). J Comput Aided Mol Des.

[56] Ryde U, Olsson MHM (2001). Int J Quantum Chem.

[57] Reuter N, Dejaegere A, Maigret B, Karplus M (2000). J Phys Chem A.

[58] Hu L, Söderhjelm P, Ryde U (2011). J Chem Theory Comput.

[59] Svensson M, Humbel S, Froese RDJ, Matsubara T, Sieber S, Morokuma K (1996). J Phys Chem.

[60] TURBOMOLE 7.0, 2015, developed by University of Karlsruhe and Forschungszentrum Karlsruhe GmbH, 1989–2015, TURBOMOLE GmbH; http://www.turbomole.com

[61] Tao J, Perdew JP, Staroverov VN, Scuseria GE (2003). Phys Rev Lett.

[62] Schäfer A, Horn H, Ahlrichs R (1992). J Chem Phys.

[63] Grimme S, Antony J, Ehrlich S, Krieg H (2010). J Chem Phys.

[64] Maier JA, Martinez C, Kasavajhala K, Wickstrom L, Hauser KE, Simmerling C (2015). J Chem Theory Comput.

[65] Hu L, Eliasson J, Heimdal J, Ryde U (2009). J Phys Chem A.

[66] Klamt A, Schüürmann J (1993). J Chem Soc Perkin Trans.

[67] Schäfer A, Klamt A, Sattel D, Lohrenz JCW, Eckert F (2000). Phys Chem Chem Phys.

[68] Sierka M, Hogekamp A, Ahlrichs R (2003). J Chem Phys.

[69] Grimme S, Ehrlich S, Goerigk L (2011). J Comput Chem.

[70] dftd3 software http://toc.uni-muenster.de/DFTD3/getd3html

[71] Weigend F, Ahlrichs R (2005). Phys Chem Chem Phys.

[72] Jensen F (1999). Introduction to computational chemistry.

[73] Kongsted J, Ryde U (2009). J Comput Aided Mol Des.

[74] Genheden S, Kuhn O, Mikulskis P, Hoffmann D, Ryde U (2012). J Chem Inf Model.

[75] Kaukonen M, Söderhjelm P, Heimdal J, Ryde U (2008). J Phys Chem B.

[76] Klamt A (1995). J Phys Chem.

[77] Eckert F, Klamt A (2002). AIChE J.

[78] Eckert F, Klamt A (2010) COSMOtherm, Version C30, Release 1301, COSMOlogic GmbH & Co KG, Leverkusen (Germany)

[79] Jorgensen WL, Chandrasekhar J, Madura JD, Impey RW, Klein ML (1983). J Chem Phys.

[80] Wang JM, Wolf RM, Caldwell KW, Kollman PA, Case DA (2004). J Comput Chem.

[81] Bayly CI, Cieplak P, Cornell WD, Kollman PA (1993). J Phys Chem.

[82] Besler BH, Merz KM, Kollman PA (1990). J Comput Chem.

[83] Frisch MJ, Trucks GW, Schlegel HB, Scuseria GE, Robb MA, Cheeseman JR, Scalmani G, Barone V, Mennucci B, Petersson GA, Nakatsuji H, Caricato M, Li X, Hratchian HP, Izmaylov AF, Bloino J, Zheng G, Sonnenberg JL, Hada M, Ehara M, Toyota K, Fukuda R, Hasegawa J, Ishida M, Nakajima T, Honda Y, Kitao O, Nakai H, Vreven T, Montgomery Jr, JA, Peralta JE, Ogliaro F, Bearpark M, Heyd JJ, Brothers E, Kudin KN, Staroverov VN, Kobayashi R, Normand J, Raghavachari K, Rendell A, Burant JC, Iyengar SS, Tomasi J, Cossi M, Rega N, Millam JM, Klene M, Knox JE, Cross JB, Bakken V, Adamo C, Jaramillo J, Gomperts R, Stratmann RE, Yazyev O, Austin AJ, Cammi R, Pomelli C, Ochterski JW, Martin RL, Morokuma K, Zakrzewski VG, Voth GA, Salvador P, Dannenberg JJ, Dapprich S, Daniels AD, Farkas Ö, Foresman JB, Ortiz JV, Cioslowski J, Fox DJ (2009) Gaussian, Inc., Wallingford CT

[84] Seminario JM (1996). Int J Quantum Chem.

[85] Nilsson K, Lecerof D, Sigfridsson E, Ryde U (2003). Acta Crystallogr D Biol Crystallogr.

[86] Tembe BL, McCammon JA (1984). J Comp Chem.

[87] Shirts MR, Chodera JD (2008). J Chem Phys.

[88] Shirts MR, Chodera JD, Python implementation of the multistate Bennett acceptance ratio (MBAR) method; http://github.com/choderalab/pymbar

[89] Bennett CH (1976). J Comput Phys.

[90] Kirkwood JG (1935). J Chem Phys.

[91] Zwanzig RW (1954). J Chem Phys.

[92] Steinbrecher T, Mobley DL, Case DA (2007). J Chem Phys.

[93] Steinbrecher T, Joung I, Case DA (2011). J Comp Chem.

[94] Kaus JW, Pierce LT, Walker RC, McCammon JA (2013). J Chem Theory Comput.

[95] Wu X, Brooks BR (2003). Phys Lett.

[96] Berendsen HJC, Postma JPM, van Gunsteren WF, Dinola A, Haak JR (1984). J Chem Phys.

[97] Darden T, York D, Pedersen L (1993). J Chem Phys.

[98] Ryckaert JP, Ciccotti G, Berendsen HJC (1977). J Comput Phys.

[99] Ross GA, Bodnarchuk MS, Essex JW (2015). J Am Chem Soc.

[100] Bodnarchuk M, Bradshaw R, Cave-Ayland A, Genheden S, Martinez AC, Michel J, Ross GA, Woods CJ, ProtoMS, School of Chemistry, University of Southampton: Southampton, U.K.; www.protoms.org

[101] Adams D (1974). J Mol Phys.

[102] Brown SP, Muchmore SW, Hajduk PJ (2009). Drug Discov Today.

[103] Bhattacharyya A (1943). Bull Cal Math Soc.

[104] Wu D, Kofke DA (2005). J Chem Phys.

[105] Pohorille A, Jarzynski A, Chipot A (2010). J Chem Phys B.

[106] Chakrabarti P, Bhattacharyya R (2007). Progr Biophys Mol Biol.

[107] Duan G, Smith VH, Weaver DF (2000). J Phys Chem A.

[108] Caldararu O, Olsson M, Riplinger C, Neese F, Ryde U (2016) J Comput Aided Mol Des (in press) 10.1007/s10822-016-9957-5 PMC5239813 27600554

[109] Laio A, Parrinello M (2002). Proc Natl Acad Sci USA.

[110] Woods CJ, Essex JW, King MA (2003). J Phys Chem B.

[111] Hamelberg D, Mongan J, McCammon JA (2004). J Chem Phys.

[112] Liu P, Kim B, Friesner RA, Berne BJ (2005). Proc Natl Acad Sci USA.

[113] Zheng L, Yang W (2012). J Chem Theory Comput.

[114] Wang L, Friesner RA, Berne BJ (2011). J Phys Chem B.

