# Supplementary material for: Binding-affinity predictions of HSP90 in the D3R Grand Challenge 2015 with docking, MM/GBSA, QM/MM, and free-energy simulations
Source: J Comput Aided Mol Des. 2016 Aug 26;30(9):707–30. doi: 10.1007/s10822-016-9942-z (PMC5078160; doi:10.1007/s10822-016-9942-z)

Binding-affinity predictions of HSP90 in the D3R Grand Challenge 2015 with docking, MM/GBSA, QM/MM, and free-energy simulations

Supplementary material

Majda Misini Ignjatović,[a] Octav Caldararu,[a] Geng Dong,[a] Camila Muñoz-Gutierrez,[b] Francisco Adasme-Carreño,[b] Ulf Ryde *[a]

[a] Department of Theoretical Chemistry, Lund University, Chemical Centre, P. O. Box 124, SE-221 00 Lund, Sweden

[b] Centro de Bioinformática y Simulación Molecular, Facultad de Ingeniería, Universidad de Talca, 2 Norte 685, Talca, Chile

2016-08-03

GCMC results

Figure S3 shows the average number of inserted water molecules at different Adams values [1], as the curve of best fit for all ligands from the set 2. The area under the curve was used to calculate the free energy to insert a number of water molecules into the binding site, using grand canonical integration [2]. It gives the free energy of transfer of a number of water molecules from the ideal gas phase to the binding site of interest. The binding free energy of a number of water molecules to the region of interest was then calculated as a difference between the free energy of transfer and the hydration free energy of the same number of water molecules. The energy profiles are shown in Figure S4.

For each protein–ligand complex, the minimum binding free energies, as well as the optimal number of water molecules and the corresponding Adams value are listed in the Table S6. The simulations at the Adams values closest to the optimum were used to cluster water positions using average linkage hierarchical clustering with a 2 Å distance cutoff. The clusters are shown in Figure 6 in the main article.

1. Adams, D.; Chemical potential of hard-sphere fluids by Monte Carlo methods; J. Mol. Phys. 1974, 28, 1241.
2. Ross G. A., Bodnarchuk M. S., Essex J. W.; Water Sites, Networks, And Free Energies with Grand Canonical Monte Carlo; J Am Chem Soc 2015, 137, 14930-14943

Table S1. Added angle parameters for three ligands, viz. the harmonic force constant (*k* in kcal/mol/rad^2) and the ideal angle (α in °).

Ligand	Atom types	*k*	α
**81**	cp-ca-cp	139.895	118.808
	c -ca-cp	184.941	121.971
**84**	cp-ca-cp	131.421	119.777
	os-ca-cp	150.926	118.705
**28**	os-ca-cp	158.983	119.651

Table S2. Added dihedral parameters for the ligands, viz. the force constant (*k* in kcal/mol/rad^2), the phase angle (φ in °), and the periodicity (*n*).

Ligand	Atom types	*k*	φ	*n*
all set1[a]	ca-hn-nh-hn	10.000	180	2
**15**	ca-ce-c3-hc	0.520	180	6
	c3-ce-ca-ca	9.742	180	2
	c2-ce-c3-hc	0.374	180	6
	c2-ce-ca-ca	5.909	180	2
**80**	h4-cd-os-cd	5.671	180	2
	cc-cc-ca-ca	5.452	180	2
	cd-cc-ca-ca	5.885	180	2
	cd-cc-ca-nb	6.189	180	2
	cc-cd-os-cd	14.226	180	2
	cc-cc-ca-nb	5.693	180	2
**81**	ca-cp-nb-ca	13.277	180	2
	cp-cp-nb-ca	16.687	180	2
**84**	ca-cp-nb-ca	12.458	180	2
	cp-cp-nb-ca	14.274	180	2

[a] Not employed in the submitted calculations in Table S5.

Table S3. Dihedral angles of the variable ring in the docked structures as well as the average angle in the FES simulations, starting from the conformation in Figure 1 (in the main text; Conf. 1) or in the flipped conformation (Conf. 2).

Ligand	Docking	Conf. 1	Conf. 2
Set 1			
**80**	107	178±7	-2±7
**81**	-70	-50±9	108±19
**82**	-70	-61±9	92±13
**83**	102	131±11	-123±19
**84**	114	123±9	-67±14
Set 3			
**10**	-136	-133±9	-42±8
**15**	-123	43±8	-126±8
**21**	-120	52±9	58±11
**23**	62	62±8	-121±7
**26**	-124	43±8	-115±9
**28**	57	49±8	50±9
**34**	-129	44±8	-130±9

Table S4. Calculated relative binding free-energies and standard errors (kJ/mol) for the 39 perturbations studied, calculated with MBAR, BAR, TI and EA methods.

Transformation			MBAR	BAR	TI	EA
Set 1	Conf. 1	ref→**80**	1.8 ± 0.5	2.0 ± 0.4	2.4 ± 1.5	2.5 ± 0.9
		**81**→**82**	-13.2 ± 0.5	-13.0 ± 0.4	-13.1 ± 1.3	-13.7 ± 0.7
		**82**→ref	13.3 ± 0.3	12.2 ± 0.3	12.0 ± 1.0	12.7 ± 0.4
		**83**→ref	3.6 ± 0.5	3.7 ± 0.4	3.7 ± 1.3	3.4 ± 0.5
		**84**→ref	8.3 ± 0.5	8.3 ± 0.4	8.4 ± 1.4	8.6 ± 0.6
Set 1	Conf. 2	ref→ **80**	-3.6 ± 0.5	-3.6 ± 0.4	-3.3 ± 1.5	-4.6 ± 0.8
		**81**→**82**	-16.4 ± 0.5	-17.7 ± 0.4	-18.1 ± 1.5	-19.1 ± 0.8
		**82**→ref	16.4 ± 0.3	16.6 ± 0.3	16.5 ± 1	15.4 ± 0.4
		**83**→ref	-3.5 ± 0.5	-2.9 ± 0.4	-2.2 ± 1.5	-2.6 ± 0.6
		**84**→ref	8.3 ± 0.6	9.1 ± 0.4	9.1 ± 1.5	8.8 ± 0.7
Set 2	2WI7	**101**→**100**	-12.2 ± 0.5	-12.1 ± 0.4	-11.8 ± 1.3	-11.7 ± 0.8
		**101**→**105**	-7.5 ± 0.2	-7.1 ± 0.2	-6.9 ± 0.8	-7.5 ± 0.4
		**101**→**106**	-7.3 ± 0.3	-6.8 ± 0.2	-6.7 ± 0.8	-7.5 ± 0.4
	3FT5	**101**→**100**	2.7 ± 0.5	2.3 ± 0.4	2.0 ± 1.4	1.8 ± 0.9
		**101**→**105**	2.7 ± 0.2	3.0 ± 0.2	3.1 ± 0.7	3.0 ± 0.3
		**101**→**106**	3.8 ± 0.3	3.6 ± 0.2	3.7 ± 0.8	3.4 ± 0.4
	2WI7/ 3FT5	**101**→**100**	-12.8 ± 0.5	-13.1 ± 0.4	-12.8 ± 1.3	-13.0 ± 0.7
		**101**→**105**	-8.4 ± 0.2	-7.9 ± 0.2	-7.8 ± 0.8	-7.8 ± 0.3
		**101**→**106**	-8.7 ± 0.3	-9.2 ± 0.3	-9.2 ± 0.9	-9.0 ± 0.4
	2WI7 +Wat2	**101**→**100**	11.2 ± 0.9	10.8 ± 0.7	11.2 ± 2.4	10.5 ± 1.3
		**101**→**105**	-6.2 ± 0.4	-5.5 ± 0.3	-5.4 ± 1.2	-5.1 ± 0.6
		**101**→**106**	-3.7 ± 0.5	-4.1 ± 0.4	-3.6 ± 1.5	-3.6 ± 0.8
	3FT5 +Wat1	**101**→**100**	18.0 ± 0.9	18.1 ± 0.7	17.9 ± 2.2	15.9 ± 1.3
		**101**→**105**	3.5 ± 0.4	3.2 ± 0.3	3.3 ± 1.2	3.5 ± 0.5
		**101**→**106**	5.5 ± 0.5	5.3 ± 0.4	5.3 ± 1.4	5.1 ± 0.6
Set 3	Conf. 1	**10**→ref 2	-4.9 ± 0.4	-4.9 ± 0.3	-5.0 ± 1.0	-5.3 ± 0.5
		**11**→ref 2	2.3 ± 0.2	1.9 ± 0.2	2.0 ± 0.7	1.2 ± 0.2
		**15**→ref 3	4.8 ± 0.5	4.7 ± 0.4	4.6 ± 1.4	1.8 ± 0.6
		**19**→ref 1	2.9 ± 0.6	2.8 ± 0.4	2.7 ± 1.5	2.9 ± 0.7
		**21**→ref 3	7.3 ± 0.4	5.7 ± 0.3	5.7 ± 1.1	6.9 ± 0.4
		**23**→ref 2	-6.7 ± 0.5	-6.9 ± 0.4	-7.1 ± 1.5	-7.0 ± 0.7
		**26**→ref 2	3.7 ± 0.4	3.7 ± 0.3	3.7 ± 1.0	3.3 ± 0.4
		**28**→ref 2	1.3 ± 0.4	1.2 ± 0.3	1.2 ± 1.1	1.1 ± 0.5
		**34**→ref 2	-0.2 ± 0.7	-0.9 ± 0.6	-1.5 ± 1.9	-9.8 ± 1.5
		**61S**→ref 2	-4.8 ± 0.8	-4.1 ± 0.6	-3.7 ± 2.1	-4.4 ± 1.4
		**61R**→ref 2	-19.8 ± 0.4	-19.7 ± 0.4	-22.3 ± 1.5	-32.0 ± 7.5
		ref 2→ref 1	-11.5 ± 0.6	-11.7 ± 0.5	-11.8 ± 1.7	-11.1 ± 0.8
		ref 3→ref 2	4.5 ± 0.4	4.6 ± 0.3	4.6 ± 1.0	4.4 ± 0.4
	Conf. 2	**10**→ref 1	-0.6 ± 0.4	-0.6 ± 0.3	-1.3 ± 1.0	-2.1 ± 0.4
		**15**→ref 3	-4.1 ± 0.6	-4.3 ± 0.4	-5.3 ± 1.6	-4.8 ± 0.7
		**21**→ref 3	-2.1 ± 0.4	-6.3 ± 0.4	-6.6 ± 1.2	-5.4 ± 0.6
		**23**→ref 2	-13.1 ± 0.6	-15.6 ± 0.5	-16.9 ± 1.5	-19.4 ± 0.7
		**26**→ref 2	-12.0 ± 0.4	-17.4 ± 0.4	-17.4 ± 1.2	-19.2 ± 0.5
		**28**→ref 2	-1.9 ± 0.4	-1.9 ± 0.3	-2.1 ± 1.1	-1.9 ± 0.4
		**34**→ref 2	-3.7 ± 0.7	-4.5 ± 0.6	-4.8 ± 1.9	-9.0 ± 1.4

Table S5. Original (submitted) relative binding free energies (obtained with MBAR in kJ/mol) for set 1, based on simulations without the improper ca–hn–nh–hn dihedral angle in Table S2, giving spurious structures, shown in Figure S2. In all cases, conformation 1 was found to be more favourable (marked in bold face).

Transformation	Conf. 1	Conf. 2
ref→ **80**	**2.7±0.4**	14.1±0.7
**81**→82	**-6.7±0.5**	-5.8±0.5
**82**→ref	**4.4±0.4**	0.4±0.4
**83**→ref	**4.1±0.5**	2.8±0.5
**84**→ref	**-1.6±0.6**	-2.1±0.6

Table S6. Minimum binding free energies (*E* in kJ/mol), the optimal number of water molecules (*N*), and the corresponding Adams value (*B*) for each protein–ligand complex studied with GCMC. Reported errors are over 1000 bootstrap samples.

Structure	Ligand	*E*	*N*	*B*
2WI7	**100**	-163.6±15.5	16.0±0.6	-7.7±0.2
	**101**	-107.5±18.8	16.8±0.8	-7.5±0.3
	**105**	-223.0±23.8	17.3±0.7	-7.8±0.3
	**106**	-161.5±22.2	17.5±0.8	-7.3±0.4
3FT5	**100**	-174.9±23.0	10.7±0.6	-7.8±0.4
	**101**	-89.1±15.1	10.9±0.6	-8.0±0.4
	**105**	-87.0±12.1	11.0±0.5	-8.0±0.3
	**106**	-73.2±12.1	10.3±0.6	-8.3±0.3

Figure S1. Ligands in the PDB structures employed.

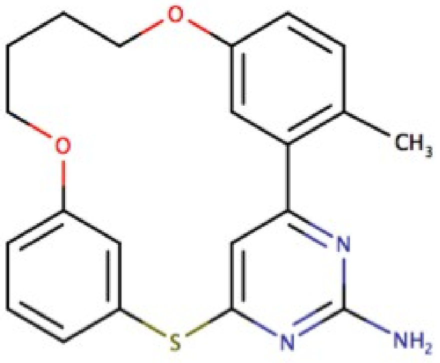

3VHA

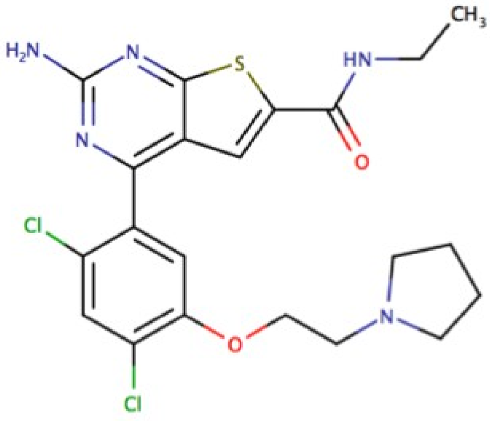

2WI7

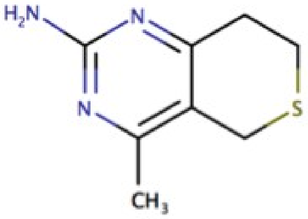

3FT5

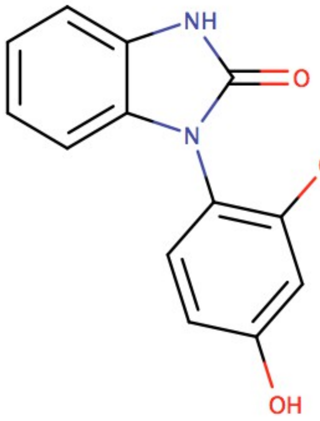

3OW6

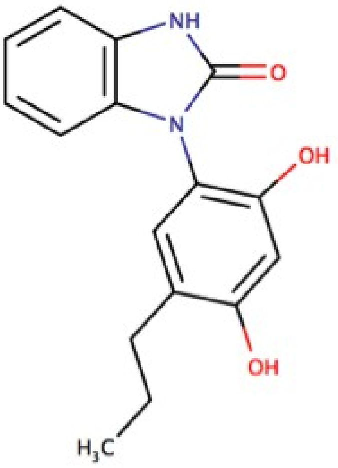

4YKR

Figure S2. Structures of the set 1 ligands obtained without the ca-hn-nh-hn improper torsion parameter shown in Table S2 (Ligands **80**, **81**, and **83** to left and ligand **82** to the right). The submitted FES results for set 1 were based on these structures.

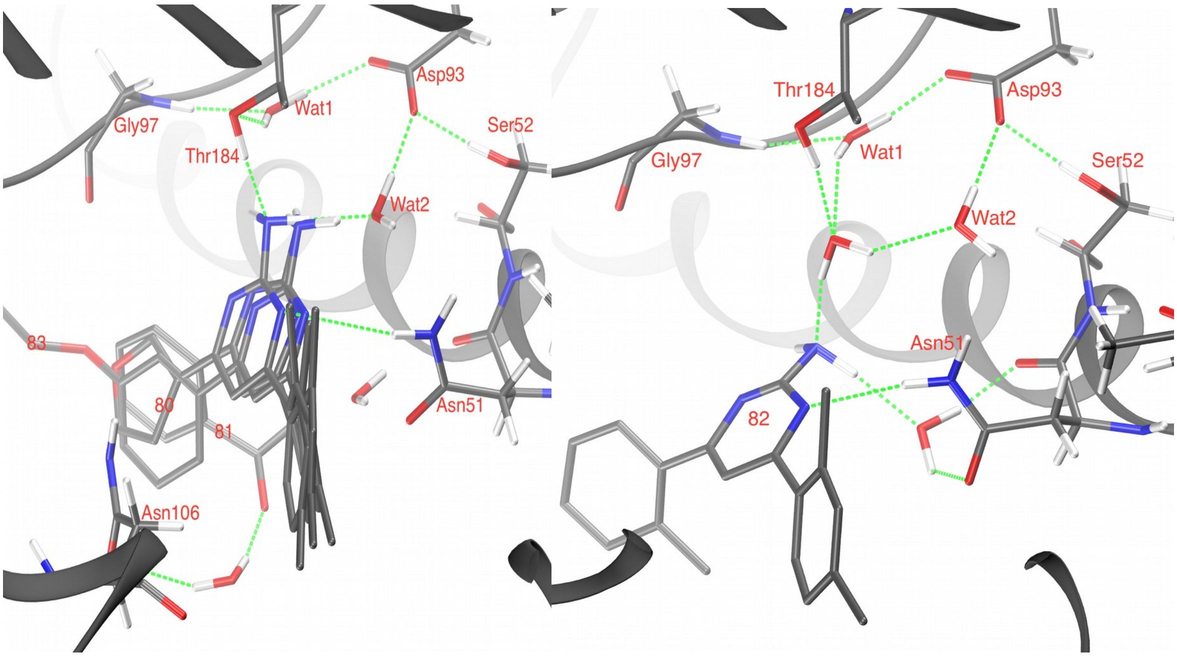

Figure S3. The average number of inserted water molecules at different Adams values, with the line of best fit, for the 2WI7 and 3FT5 complexes with set2 ligands. The light orange area shows where 50% of the bootstrap samples produced the line of best fit, and the light grey where 90% of the fits lay.

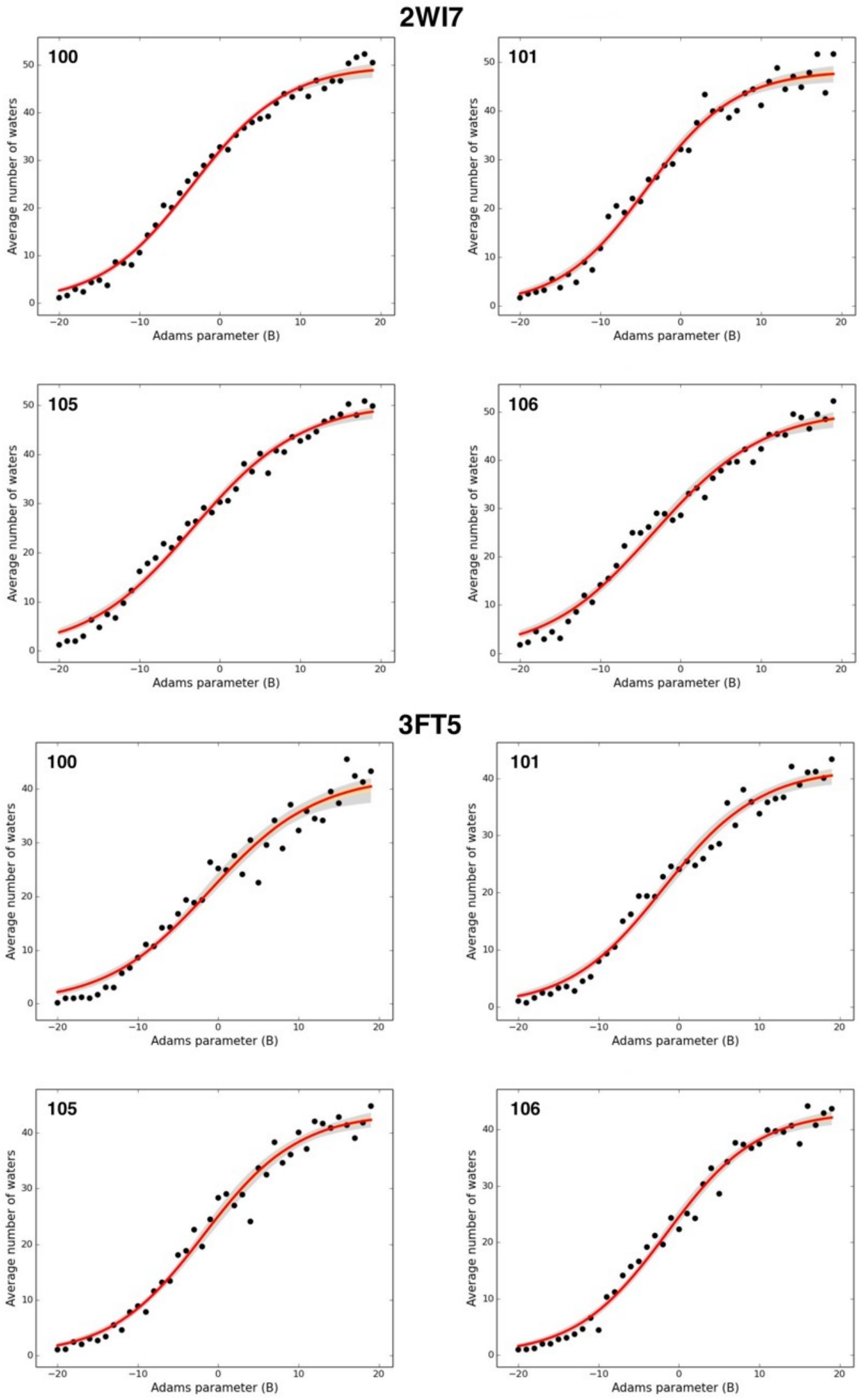

Figure S4. Free energies of binding water molecules into the binding sites of 2WI7 and 3FT5 complexes with the set 2 ligands. Errors were calculated using bootstrap sampling of the GCMC titration data (1000 bootstraps); the light blue areas show the top 50% of the bootstrapped free energies and the light grey areas show the top 90%.

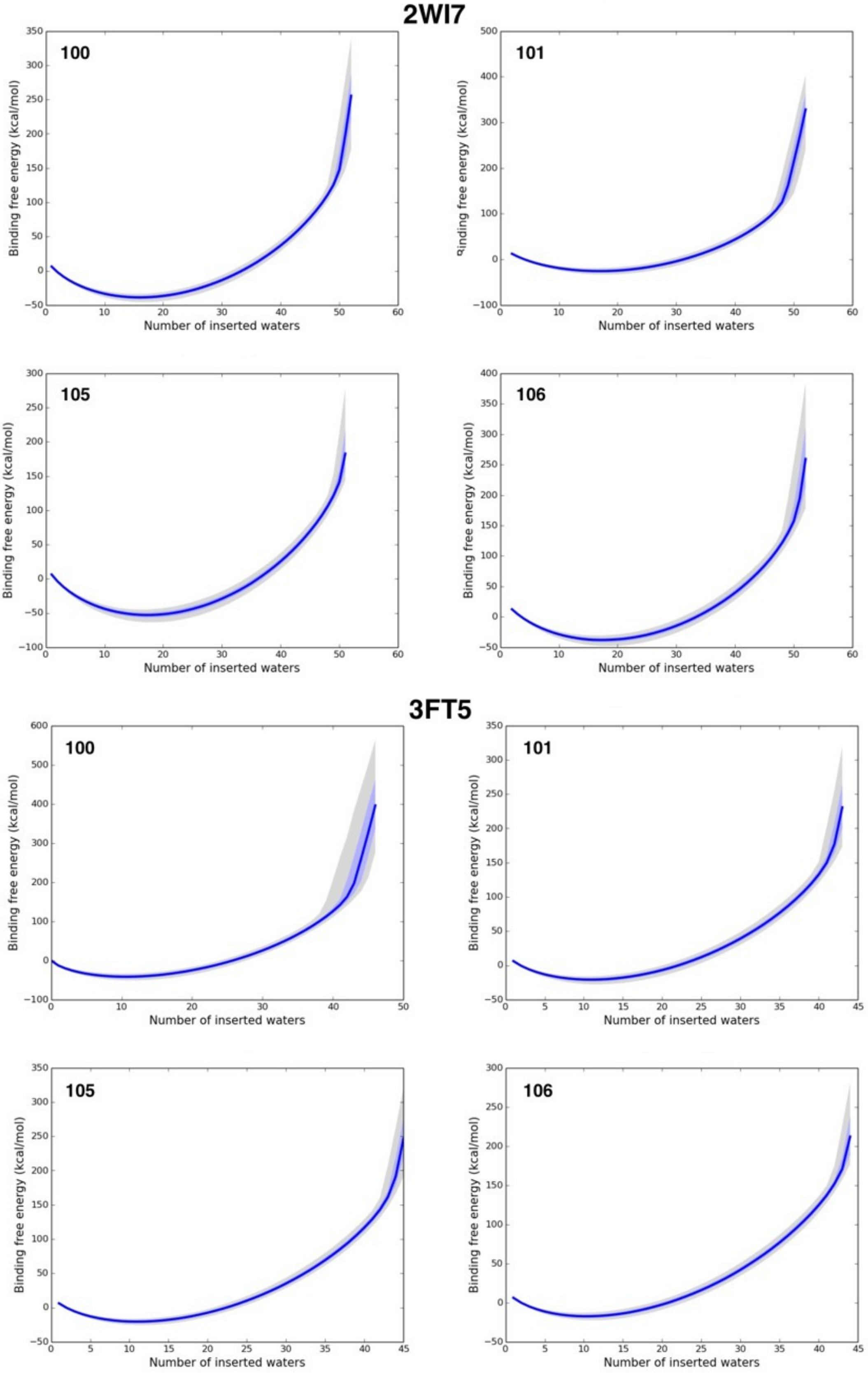

Supplement: Supplementary file 1 — Supplementary material 1 (PDF 598 kb) [file 10822_2016_9942_MOESM1_ESM.pdf]
